# An investigation into per- and polyfluoroalkyl substances (PFAS) in nineteen Australian wastewater treatment plants (WWTPs)

**DOI:** 10.1016/j.heliyon.2019.e02316

**Published:** 2019-08-23

**Authors:** Timothy L. Coggan, Damien Moodie, Adam Kolobaric, Drew Szabo, Jeff Shimeta, Nicholas D. Crosbie, Elliot Lee, Milena Fernandes, Bradley O. Clarke

**Affiliations:** a Centre for Environmental Sustainability and Remediation, School of Science, RMIT University, GPO Box 2476, Melbourne, Vic., 3001, Australia; b Applied Research, Melbourne Water Corporation, Docklands, VIC, 3001, Australia; c Faculty of Engineering, University of New South Wales, NSW, 2052, Australia; d Water Corporation, Leederville, Western Australia, 6007, Australia; e SA Water, GPO Box 1751, Adelaide SA, 5001, Australia; f College of Science and Engineering, Flinders University, Adelaide, Australia

**Keywords:** Environmental science, Chemistry, Chromatography, Environmental analysis, Environmental assessment, Environmental chemistry, Environmental hazard, Waste, Environmental pollution, Water pollution F–53B, FTS, Wastewater, Sludge, PFAS, PFOA, PFOS

## Abstract

Quantifying the emissions of per- and polyfluoroalkyl substances (PFAS) from Australian wastewater treatment plants (WWTP) is of high importance due to potential impacts on receiving aquatic ecosystems. The new Australian PFAS National Environmental Management Plan recommends 0.23 ng L^−1^ of PFOS as the guideline value for 99% species protection for aquatic systems. In this study, 21 PFAS from four classes were measured in WWTP solid and aqueous samples from 19 Australian WWTPs. The mean ∑_21_PFAS was 110 ng L^−1^ (median: 80 ng L^−1^; range: 9.3–520 ng L^−1^) in aqueous samples and 34 ng g^−1^ dw (median: 12 ng g^−1^ dw; range: 2.0–130 ng g^−1^ dw) in WWTP solids. Similar to WWTPs worldwide, perfluorocarboxylic acids were generally higher in effluent, compared to influent. Partitioning to solids within WWTPs increased with increasing fluoroalkyl chain length from 0.05 to 1.22 log units. Many PFAS were highly correlated, and PCA analysis showed strong associations between two groups: odd chained PFCAs, PFHxA and PFSAs; and 6:2 FTS with daily inflow volume and the proportion of trade waste accepted by WWTPs (as % of typical dry inflow). The compounds PFPeA, PFHxA, PFHpA, PFOA, PFNA, and PFDA increased significantly between influent and final effluent. The compounds 6:2 FTS and 8:2 FTS were quantified and F–53B detected and reported in Australian WWTP matrices. The compound 6:2 FTS was an important contributor to PFAS emissions in the studied Australian WWTPs, supporting the need for future research on its sources (including precursor degradation), environmental fate and impact in Australian aquatic environments receiving WWTP effluent.

## 1 Introduction

Per- and polyfluoroalkyl substances (PFAS) are a group of man-made pollutants that pose an emerging risk to the water sector, challenging established practices such as recycling and environmental discharges. They are omnipresent in water, air, food, wildlife, and humans, are resistant to typical environmental degradation processes, and can have negative impacts on exposed organisms [1, 2, 3, 4, 5, 6, 7, 8, 9, 10]. Most PFAS are recalcitrant through conventional water treatment processes and, therefore, wastewater effluents can contain PFAS that has originated from domestic and industrial sources [11, 12]. Understanding the sources of PFAS to the environment is of high importance in Australia due to the recently recommended perfluorooctane sulfonic acid (PFOS) guideline value for 99% species protection of 0.23 ng L^−1^ in aquatic ecosystems in the PFAS National Environmental Management Plan [13].

The unique and useful chemical and physical properties of PFAS have resulted in many commercial applications, such as stain-resistant coatings, water-resistant fabrics, metal plating paints, pesticides, fluoropolymers, greaseproof paper, and aqueous film-forming foams (AFFF) used in firefighting, amongst others [3, 4, 7, 9]. Although PFAS are a broad class of compounds comprising over 4700 known PFAS [10], many studies have focused on a small number of perfluoroalkyl acids (PFAAs), specifically the perfluorocarboxylic acids (PFCAs; CF_3_(CF_2_)_n_COOH) and perfluorosulfonic acids (PFSAs; CF_3_(CF_2_)_n_S(=O)OH) [9]. Despite being manufactured since the 1950s, it wasn't until 2001 that the extent of PFAS global contamination was first demonstrated for perfluorooctane sulfonate (PFOS; C_8_F_17_SO_3_H) and perfluorooctanoic acid (PFOA; C_7_F_15_COOH) [1]. Since then, PFAS have been detected in almost every wildlife sample measured [14], ubiquitously in humans throughout the world [15], and in most environmental compartments, including pristine locations [7].

The perfluoroalkyl substances contain at least one fully fluorinated alkyl chain bonded to a functional group, whereas polyfluoroalkyl substances contain a partially fluorinated alkyl chain with a range of functional groups. In general, the sorption potential of PFAS is determined by functional group, chemical structure, and fluorinated chain length; however, for many newer PFAS, this information is not yet available. In environmental aquatic systems, the different partitioning behavior will typically result in short-chain compounds (PFCAs: ≤ C6, and PFSAs: ≤ C5) partitioning to the aqueous phase and long-chain compounds adsorbed to the solid compartments [16, 17]. Furthermore, some PFAS (viz. fluorotelomer alcohols, phosphate esters, etc.) are precursor compounds and will transform in the environment, forming many intermediate transformation products with PFAAs such as PFOA as terminal products [18].

The growing understanding of the risks of many legacy PFAS has led to the phase-out of production of PFOS (and related compounds) and PFOA in North America (in 2000 and 2002, respectively) and an increased use of less problematic alternative compounds (such as short-chain and fluorotelomer based chemistries) [4]. An example of two PFOS alternatives used as mist suppressants in metal plating are 6:2 fluorotelomer sulfonate (6:2 FTS) and the chlorinated perfluoroether sulfonate F–53B [4, 19, 20]. In some regions, 6:2 FTS is not used as a PFOS substitute in metal plating as it cannot match the low surface tension of PFOS and approximately three to ten times the quantity is required [21]. However, 6:2 FTS has found further uses as a PFOS substitute in AFFF, oil production and primarily occurs as an intermediate degradant of complex fluorotelomer-based substances [4]. In initial testing by Dupont scientists, 6:2 FTS was found to show low risk to aquatic ecosystems making it a desirable substitute, however, studies on the environmental fate and effects were still needed [22]. Alternatively, F-53 (6:2 PFESA) then the chlorine substituted F–53B (6:2 Cl-PFESA), have been used almost exclusively in China since the 1970s with little PFOS ever used in metal plating [19]. As investigations into the fate and toxicity of F–53B progresses, it is now becoming apparent that it shows similar recalcitrance, toxicity and physiochemical properties to PFOS and is becoming widely distributed in the environment making it a less desirable substitute for PFOS [20, 23, 24, 25].

Wastewater treatment plants (WWTPs) can act as a conduit for many recalcitrant anthropogenic compounds, such as PFAS, to the environment through effluent discharges and the land application of biosolids [26]. PFAS have been detected in WWTP influent, effluent and solids worldwide [11]. Similar to other environmental compartments, hydrophobic partitioning in WWTPs is the dominant sorption mechanism, which results in long-chain PFAAs partitioning to WWTP solid matrices [27, 28, 29, 30]. Typical wastewater treatment processes are unable to remove PFAS from the final effluent. In some studies, concentrations of compounds such as perfluorocarboxylic acids (PFCA) and perfluorosulfonic acids (PFSA) have increased from influent to final effluent [11, 27, 31]. The increase of PFAAs has been attributed to the degradation of the PFAS precursor compounds [32, 33], fluorotelomer sulfonoates (FTS) and fluorotelomer alcohols (FOTH), that have been shown to transform to stable PFAAs in WWTP sludge [34, 35].

The awareness of PFAS environmental contamination associated with AFFF application on government military sites, and evidence of widespread distribution in the Australian environment [36, 37, 38, 39, 40], have led to the development of the Australian PFAS National Environmental Management Plan (NEMP) [13]. Within the NEMP, the recommended freshwater and marine guideline values (water concentrations) for 99% species protection are 0.23 and 1900 ng L^−1^ PFOS and PFOA, respectively (HEPA 2018). As a result, there is strong interest from water industry professionals and regulators to understand the quantities of PFAS released into the environment through treated effluent, and the potential impact these emissions may have upon Australian aquatic environments.

Initial studies on PFAS emissions in Australian WWTPs have focused on the removal efficiency in two reclaimed water plants (18 PFAS measured ranging from 1.1 to 38.6 ng L^−1^) [41] and one WWTP (8 PFAS measured ranging from 3 to 82 ng L^−1^) [42]. An Australian-wide study measuring nine PFAS in WWTP effluent (range from n. d. to 240 ng L^−1^) and biosolids, sampled in 2016, estimated that Australian WWTPs have discharged an estimated 33 kg PFOS and 67 kg PFOA, annually [37]. More recently, PFAS levels in influent over a four year period at two large Australian WWTPs (mean ∑_11_PFAS levels 57 ± 3.3–94 ± 17 ng L^−1^ at WWTP A; and 31 ± 6.1–142 ± 73 ng L^−1^ at WWTP B) were determined to have: 1) no significant difference in daily PFAS mass load between weekdays and weekends (composite samples over 7 consecutive days), 2) very few significant seasonal differences of ∑_11_PFAS (with most significant differences linked to a pulse release of PFOS at both WWTPs), and, 3) only one significantly different annual mean mass load in WWTP B over the entire four year period (linked to the same PFOS pulse event of October 2017) [43].

Australian WWTPs represent a unique case as there is no reported PFAS manufacture and low rates of PFAS are imported for direct use in industries such as car manufacture, chrome plating, leather treatment, medical imaging, firefighting and in goods already impregnated (carpets, furniture, etc.) or in products containing PFAS as impurities [13, 44]. Furthermore, unlike many parts of the world, in Australian cities, sewer systems are closed, with separate stormwater sewers and low infiltration rates, this means rainfall has limited effect on influent PFAS composition as opposed to pulse events from industrial effluent discharge. It is, however, becoming apparent that many PFAS, including PFOS and PFOA, are present in Australian WWTP effluents and are being discharged to the aquatic environment.

The aims of this study were to measure the mass loading of PFAS (including PFAAs, FTSs, and F–53B) within solid and liquid matrices from 19 Australian WWTPs of varying size, capacity, localities and treatment types. Samples were taken from various stages within the treatment train from a range of WWTPs to determine the trends in the mass flux and partitioning of PFAS within the sampled WWTPs. Finally, the data were compared to recent work estimating the Australian annual PFAS discharge, providing important data for ongoing assessments of the potential impact of PFAS on aquatic environments.

## 2 Materials and methods

### 2.1 Sampling

Field sampling kits including field blanks were prepared at RMIT University laboratories and shipped overnight to each WWTP. Three replicate aqueous and solid samples were collected from each of nineteen Australian WWTPs throughout 2017 (Table 1). Aqueous samples (influent, primary effluent, secondary effluent, final effluent, recycled water) consisting of either triplicate sub-samples from a single 24 h composite or three replicate grab samples were collected in 250 mL polypropylene bottles pre-rinsed with ultrapure water, methanol, and site water. Solid samples (primary sludge, secondary sludge, lagoon sludge, and one lagoon sludge dredge pile) were collected in 50 mL polypropylene centrifuge tubes. On receipt, samples were sterilized (aqueous samples with sodium azide ∼1g L^−1^ and solid samples with 2 % w/w sodium azide solution) and refrigerated until extraction.

**Table 1:** Wastewater treatment plant specifics and sample locations for replicate influent (IN), primary effluent (1E), secondary effluent (2E), final effluent (FE) and recycled water (RW). Lagoon sludge (LS) was collected from WWTPs-8, 10 and 16; primary (1S) and secondary (2S) sludge were collected from WWTPs-3, 4, 9, 17 and 18.

WWTP code	Treatment description	Month sample	WWTP type	Inflow (ML/d)	TW (%)
WWTP-1	screen (IN), IDEA (2E), balancing pond (FE)	AUG	AS	6	<10%
WWTP-2	screen (IN), SBR, filtration, UV disinfection (FE)	APRIL	AS	13	<10%
WWTP-3	screen (IN), primary sedimentation (1E), aeration, secondary sedimentation (FE) - FE excess sludge and centrifuge supernatant to DAFT, then DAFT supernatant to aeration tanks	AUG	AS	127	<5%
WWTP-4	screen (IN), primary sedimentation (1E), activated sludge reactors, clarifier (2E), stabilisation lagoons (FE), dissolved air floatation and filtration (RW-1), chlorination (RW-F) - secondary sludge and centrifuge supernatant to activated sludge reactors	AUG	AS/LAG	167	<20%
WWTP-5	screen (IN), bioselector, SBR (2E), balancing dam (FE) - Excess aeration sludge to processing, sludge supernatant to influent	DEC	AS	9.8	<5%
WWTP-6	screen (IN), bioselector, oxidation ditches (1E), clarifiers (FE), tertiary filters, UV disinfection (RW) - Excess secondary sludge to aerated storage tanks, and centrifuge supernatant to influent	SEPT	AS	4.9	<5%
WWTP-7	screen (IN), bioselector, oxidation ditches (1E), clarifiers (FE)	OCT	AS	2.7	<5%
WWTP-8	screen (IN), aeration pond, maturation pond (FE)	DEC	LAG	1.59	<5%
WWTP-9	screen (IN), primary sedimentation (1E), aeration (2E), balancing dam, media filtration, ozone, UV disinfection, chlorination (FE)	SEPT	AS	330	<20%
WWTP-10	screen (IN), aeration pond (1E), maturation pond (FE)	OCT	LAG	1.9	<10%
WWTP-11	screen (IN), bioselector, oxidation ditches (1E), clarifiers (FE) - centrifuge supernatant to bioselector	NOV	AS	10.2	<5%
WWTP-12	(IN), screen, bioselector, SBR with alum addition (1E), balancing dam (FE), tertiary filters, chlorine disinfection (RW) - excess secondary sludge to digesters, digester and centrifuge supernatant to influent	SEPT	AS	3.2	<5%
WWTP-13	screen (IN), bioselector, oxidation ditches (1E), clarifiers (FE) - Excess secondary sludge to DAFT, DAFT and centrifuge supernatant to bioselector	NOV	AS	5.5	<5%
WWTP-14	(IN), screen, bioselector, SBR with alum addition, balancing dam (FE), tertiary filters, chlorine disinfection (RW) - excess secondary sludge to aerated storage tanks, and centrifuge supernatant to influent	DEC	AS	1.5	<5%
WWTP-15	screen (IN), Imhoff tank, primary pond, secondary ponds (2E), alum dosing, polishing pond (FE), UV disinfection (RW), chlorination	NOV	LAG	1.5	<5%
WWTP-16	(IN), screen, Anaerobic ponds (1E,1E), facultative ponds, maturation ponds (FE)	AUG	LAG	3.7	<5%
WWTP-17	screen (IN), primary sedimentation (1E), aeration, secondary sedimentation (FE) - excess secondary sludge and centrifuge supernatant to DAFT, then DAFT supernatant to primary sedimentation	SEPT	AS	59	<10%
WWTP-18	screen (IN), primary sedimentation (1E), SBR (2E), balancing dam (FE) - centrifuge supernatant and excess SBR sludge to DAFT, then DAFT supernatant to Primary sedimentation tanks	AUG	AS	143	<10%
WWTP-19	(IN) anaerobic ponds, aerobic ponds, clarifiers (2E, 2E), maturation ponds (FE), polishing pond (RW-1), UV disinfection, chlorine disinfection (RW-F)	SEPT	AS/LAG	498	<30%

WWTP treatment trains were broadly classified as activated sludge (AS) and lagoon based (LAG). TW refers to the proportion of trade waste (TW) of typical dry inflow received at the sampled WWTPs. Trade waste flows were calculated from metered flows at industrial sites, industry models or estimates of commercial discharges. The acronyms IDEA (intermittently decanted extended aeration), SBR (sequencing batch reactors) and DAFT (dissolved air floatation thickeners) refer to treatment process employed within the WWTPs.

### 2.2 Chemicals and standards

The compounds quantified in this study were the perfluorocarboxylic acids (PFCAs): PFBA, PFPeA, PFHxA, PFHpA, PFOA, PFNA, PFDA, PFUdA, PFDoA, PFTrA & PFTeA; the perfluorosulfonic acids (PFSAs): PFBS, PFPeS, PFHxS, PFHpS, PFOS, PFDS; the fluorotelomer sulfonates 6:2 FTS, 8:2 FTS, and the chlorinated perfluoroether sulfonic acids (components of the commercial product F–53B): 6:2 Cl-PFESA (F–53B) and the F–53B impurity 8:2 Cl-PFESA (full compound details and MS/MS transitions listed in Table S1). These compounds were selected as PFCAs and PFSAs have previously been demonstrated to be present in Australian WWTPs and need further baseline data [37, 41, 42]. The FTSs were selected as 6:2 FTS has been demonstrated as present in AFFF formulations impacting WWTPs [33], used as a PFOS replacement [4] and there is little current published Australian data on FTSs. Furthermore, the F–53B components are an emerging contaminant in China due to substitution for PFOS in chrome plating [20]. As Australian is part of the Asia Pacific region, and Cl-PFESAs have been detected in WWTPs in China [19, 24] it was included in this study to determine if there is an emerging risk in Australia.

Analytical standards and isotopically labeled analogues of PFAS were purchased from Wellington Laboratories (Ontario, Canada) as solutions of 50 μg mL^−1^ in methanol. Stock solutions of 100 ng mL^−1^ for native PFAS and 100 ng mL^−1^ for surrogate PFAS were prepared gravimetrically in methanol for spiking.

The solvents methanol (LC-MS grade, Honeywell, USA and LiChrosolv hypergrade, Merck Millipore, Australia) and ultrapure water (pH 8, Merck Millipore, Australia) were tested for PFAS contamination prior to use. Ammonium hydroxide solution (28% in H_2_O, ≥ 99.99%), sodium acetate, glacial acetic acid and ammonium acetate (≥99.99%) were purchased from Sigma-Aldrich (Australia). The dispersive solid-phase extraction sorbents (d-SPE), sorbents C18, and primary secondary amine (PSA) were purchased in bulk from Agilent Technologies (USA).

### 2.3 Aqueous sample extraction

Aqueous samples were extracted using similar methods outlined in Szabo, Coggan [40], Hepburn, Madden [45] and Coggan, Anumol [46]. Briefly, samples were filtered using 1 μm glass fibre filters (Merck Millipore, Australia), spiked with 5 ng of isotopically labelled PFAS, followed by solid-phase extraction (SPE) using Oasis weak anion exchange (6 mL, 150 mg WAX) cartridges with 15 mL polypropylene centrifuge vials used as collection vessels. Cartridges were conditioned sequentially with 4 mL 0.1% (v/v) ammonium hydroxide in methanol, 4 mL methanol, and 4 mL ultrapure water. The entire sample was passed through the cartridge under vacuum at approximately one drop per second, then washed with 4 mL of a pH 4 buffer (sodium acetate/acetic acid) and dried under vacuum for 10 min. SPE cartridges were eluted using 2 mL of methanol that was used to rinse the sample bottle, followed by 4 mL of 0.1% (v/v) ammonium hydroxide in methanol. Extracts were evaporated to 500 μL under a gentle stream of nitrogen (at 25 °C) and reconstituted to 1 mL in methanol and transferred to a polypropylene chromatography vial with polyethylene lid for analysis.

### 2.4 Solid sample extraction

Freeze-dried sludge samples (0.5–1 g) were spiked with 25 ng of isotopically labelled PFAS before adding 4.65 mL of 10 mM NaOH in methanol. Samples were sonicated for 30 min and shaken overnight for 12 h. Extracts were neutralized with 100 μL of glacial acetic acid and cooled on ice. Five mL of extract was then transferred to a 15 mL polypropylene (PP) tube before adding 100 mg of C18 and 50 mg primary secondary amine (PSA) to remove interfering compounds. Extracts were agitated for approximately 1 min and centrifuged (10,000 rpm, 10 °C, 10 min), with this process repeated twice. Finally, extracts were filtered using a 0.45 μm PES syringe filter (pre-rinsed with LC-MS grade methanol) into a propylene chromatography vial with polyethylene lid for analysis.

### 2.5 Instrumental analysis

The analysis was performed using liquid chromatography-tandem mass spectrometry (LC-MS/MS) on an Agilent 6495B mass spectrometer coupled with an Agilent 1290 II Infinity liquid chromatograph optimised for PFAS analysis. Twenty-one PFAS compounds were quantified using isotope dilution. A surrogate compound for each PFAS was set as a mass-labeled compound from a similar class and/or close elution time. For compounds where two or more transition ions were present, the transition with the highest response was set as the quantifier, with others set as qualifier ions. The branched plus linear isomers of PFPeS, PFHxS, PFHpS, and PFOS were quantified using linear-only calibration standards and reported as a combined branched plus linear concentration.

The twenty-one PFAS quantified in the analytical method are listed in the supplementary information (Table S1). The method employed dynamic multiple reaction monitoring (dMRM) and a 2 μL injection, in negative ESI mode. MS parameters were: gas temperature 250 °C, gas flow 11 L min^−1^, Nebulizer 25 psi, sheath gas temp 375 °C, sheath gas flow 11 L min^−1^, capillary voltage 2500 V, high pressure ion funnel RF 90 V and low pressure ion funnel RF 60 V. Separation was achieved using a Zorbax eclipse plus RRHD C18 column (3.0 × 50 mm, 1.8 μm, Agilent Technologies, USA) with a guard column attached. Gradient elution with the solvents 5 mM ammonium acetate in ultrapure water (A) and methanol (B) at 400 μL min^−1^ was performed, and the first 1.5 min was diverted to waste (t_0_ = 10% B; t_0.5_ = 10% B; t_2.5_ = 55% B; t_9_ = 90% B; t_9.5_ = 100% B; t_11.5_ = 100% B; t_11.6_ = 10% B; t_14_ = 10% B). A delay column (Zorbax Eclipse Plus C18 RRHD, 4.6 × 50 mm, 3.5 μm, Agilent Technologies, USA) was installed between the solvent mixer and injector module to delay instrument PFAS contamination.

### 2.6 Quality control

Linear calibration curves were prepared by gravimetric dilution of a mixed PFAS standard solution (100 ng mL^−1^ in methanol) with methanol to 9 levels with r^2^ > 0.99. Limit of quantification (LOQ) was set as the lowest calibration point multiplied by four and ranged from 0.1 to 0.6 ng L^−1^ depending on the compound type and gravimetric dilution. Limit of detection (LOD) was set as the instrument detection limit (IDL), varied on a compound-by-compound basis, and ranged from 0.01 to 0.1 ng L^−1^. IDL and instrument variability was determined using similar methods to Coggan, Anumol [46] on the same instrument and using the same instrument configuration.

A field blank was prepared with every kit, transferred to a clean bottle on-site and then extracted concurrently with samples. Field blanks were extracted within the same batches as samples and matched with corresponding WWTPs. Only one compound (PFBA) was detected above LOD in field blanks from treatment plants WWTP-7 and WWTP-17; due to this, PFBA results for these two treatment plants were set as < LOD.

Aqueous samples were extracted in batches containing two method blanks and a laboratory control sample (LCS). Laboratory control samples consisted of ultrapure water spiked with a native PFAS mixture containing all measured compounds at a mass of 5 ng, 1 ng or 0.25 ng. Mean recovery of all compounds in LCS samples ranged from 80 to 120% with s.d. < 15%, except for PFDS (72%, s.d. 13%), 8:2 Cl-PFESA (73%, s.d. 7%), PFTrA (70%, s.d. 6%) and PFTeA (76%, s.d. 6%) (Table S2). Solid LCS samples consisted of acid-washed sand spiked with 10 ng of PFAS and extracted alongside batches of 12 samples. Mean recovery of all compounds in LCS samples ranged from 80 to 120% with s.d. < 15%, except for 6:2 FTS (61% s.d. 8%). Method blanks returned less than the limit of detection (<LOD) for all batches. The use of ultrapure water and acid-washed sand as laboratory control samples may not adequately represent WWTP matrices (and the associated interferences) and present some uncertainty with analytical results. However, similar methods have been successfully employed in WWTP matrices in other studies [32, 46, 47] and overall we considered the QA/QC results provided an acceptable assurance of the quality of the data set for this study.

### 2.7 Data processing

Quantitation was carried out using MassHunter QQQ quantitative analysis software (version 08.00, Agilent Technologies, USA). Descriptive statistics were computed using pooled data from all samples.

Statistical analysis was carried out using R [48]. Data visualizations were also produced in R [48] using the packages reshape2 [49] and ggplot2 [50].

To compare distribution coefficients to those previously published in Eriksson, Haglund [32] and Sun, Zhang [51], similar estimation methods were employed. This calculation method is only an approximation as it assumed that the concentration of PFAS in solids and liquids at the sampled location were in equilibrium and does not consider the differences in effluent and sludge retention times. Distribution coefficients (log K_d_) were calculated for the compounds PFHxA, PFHxS, 6:2 FTS, PFOA, PFNA, PFOS, and PFDA. Distribution coefficients were only calculated for these compounds at sample locations where both aqueous and solid samples were above the limit of quantitation.

Due to non-normal distributions, data for 11 compounds were first log_10_-transformed. Pearson correlation coefficients were computed using the transformed, pooled, influent and pooled final effluent data. Linear mixed-effects analysis was performed on the transformed data using the R package lme4 [52]. P-values were obtained by likelihood ratio tests of the full model with treatment stage (influent and final effluent) included against the model without the effect in question. PCA analysis was performed on the untransformed data for the 11 compounds plus percentage trade waste and daily inflow using the correlation matrix (standardised) and visualised in R using the package factoextra [53] and ggplot2 [50].

## 3 Results and discussion

### 3.1 PFAS in WWTP matrices

Twenty-one PFAS from four classes (PFCA, PFSA, FTS, Cl-PFESA) were measured in aqueous (n = 201) and solid (n = 51) samples from the 19 Australian WWTPs. PFAS were detected in all samples from all matrices. The summary statistics are presented in Table 2 and the data is further provided in the Supplementary Information, Table S3, and Table S4.

**Table 2:** Summary statistics for pooled aqueous (n = 201, triplicates from 67 individual locations within 19 WWTPs) and pooled solid (n = 51, triplicates from 5 primary and secondary sludge locations, 6 lagoon sludges and a lagoon dredge pile) samples. The sum of branched plus linear isomers was reported for PFPeS, PFHxS, PFHpS, and PFOS.

	Aqueous samples (ng L^−1^)						Solid samples (ng g^−1^ dw)					
	Median	Mean	s.d.	min	Max	Detect (%)	Median	Mean	s.d.	min	max	Detect (%)
PFBA	5.8	13	33	<LOQ	370	100%	<LOD	0.45	0.91	<LOD	4.1	29%
PFPeA	5.3	8.3	8.8	<LOD	47	96%	<LOD	<LOQ		<LOD	5.2	20%
PFHxA	16	21	17	1.4	92	100%	0.92	1.9	2.8	<LOD	13	82%
PFHpA	5.0	6.1	5.1	<LOD	34	100%	<LOQ	0.30	0.66	<LOD	4.1	54%
PFOA	11	19	19	1.0	91	100%	<LOQ	2.6	4.4	<LOD	25	84%
PFNA	0.60	0.92	1.1	<LOD	6.6	97%	<LOQ	0.20	0.29	<LOD	1.1	50%
PFDA	1.3	2.3	2.9	<LOD	18	98%	0.60	5.1	7.7	<LOD	26	84%
PFUdA	<LOD	<LOQ		<LOD	<LOQ	12%	<LOQ	<LOQ		<LOD	1.2	54%
PFDoA	<LOD	0.47	0.55	<LOD	4.2	49%	0.48	3.8	5.9	<LOD	20	94%
PFTrA	<LOD	<LOQ		<LOD	<LOQ	19%	<LOQ	0.32	0.48	<LOD	1.8	70%
PFTeA	<LOD	0.27	0.19	<LOD	2.0	25%	<LOQ	0.69	1.1	<LOD	4.6	90%
PFBS	2.5	4.0	4.9	<LOD	33	98%	<LOD	0.83	2.0	<LOD	9.3	44%
PFPeS	<LOQ	1.9	4.1	<LOD	27	77%	<LOD	<LOQ		<LOD	2.3	14%
PFHxS	3.1	13	31	<LOD	200	95%	<LOQ	1.1	2.8	<LOD	17	50%
PFHpS	<LOQ	0.86	1.7	<LOD	11	76%	<LOD	0.29	0.67	<LOD	3.3	26%
PFOS	7.2	15	24	<LOD	140	99%	4.7	14	24	<LOD	90	94%
PFDS	<LOD	0.21	0.13	<LOD	1.1	23%	<LOD	0.78	2.1	<LOD	9.8	42%
6:2 FTS	2.4	7.3	12	<LOD	61	99%	<LOD	0.26	0.69	<LOD	2.7	26%
8:2 FTS	<LOQ	0.53	1.1	<LOD	9.2	82%	<LOD	0.73	1.6	<LOD	6.9	42%
6:2 Cl-PFESA	<LOD	<LOQ		<LOD	<LOQ	4%	<LOD	<LOQ		<LOD	<LOQ	16%
8:2 Cl-PFESA	<LOD	<LOD		<LOD	<LOD	0%	<LOD	<LOQ		<LOD	<LOQ	8%
∑_21_PFAS	80	110		9.3	520		12	34		2.0	130	

#### 3.1.1 Aqueous matrices

The mean ∑_21_PFAS in aqueous samples was 110 ng L^−1^ (median: 80 ng L^−1^; range: 9.3–520 ng L^−1^) (Table 2). The highest concentration measured in aqueous matrices for any compound was 370 ng L^−1^ for PFBA in final effluent at WWTP-12. PFBA has been used as a short-chain PFAS substitute for some PFCAs [54]. The high concentration of PFBA in final effluent and distribution within the sampled WWTPs may reflect current PFBA use.

Eleven of the 21 compounds were detected in >90% of samples (PFBA, PFPeA, PFHxA, PFHpA, PFOA, PFNA, PFDA, PFBS, PFHxS, PFOS, 6:2 FTS), and these were used in the subsequent statistical analysis of influent and final effluent. Mean concentrations in aqueous samples followed the trend: PFHxA > PFOA > PFOS > PFHxS > PFBA > PFPeA>6:2 FTS > PFHpA > PFBS > PFDA > PFPeS > PFNA > PFHpS>8:2 FTS > PFTeA > PFDS. Mean concentrations of PFAS in aqueous samples were similar to concentrations previously reported for Australian WWTP aqueous samples [37, 41, 43]. To date concentrations of PFBA and 6:2 FTS have not been widely reported in Australian WWTPs. PFBA and 6:2 FTS have been used as C8 substitutes and are end-stage and intermediate metabolites (respectively) of many PFAS, their prevalence in the studied WWTPs may be an indicator of changing PFAS use trends in Australia.

**Fig. 1:**
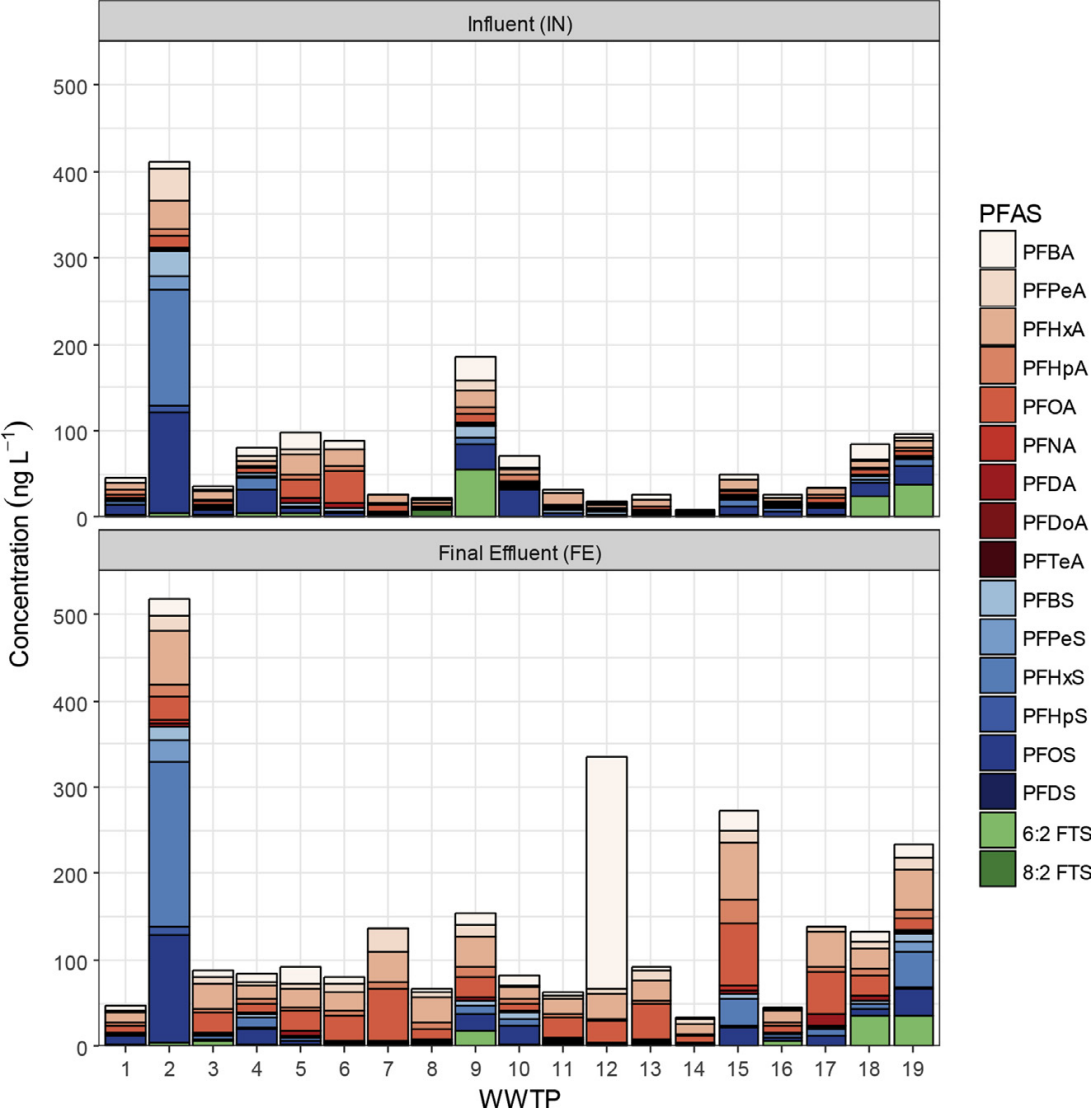
Mean PFAS concentration (n = 3 replicates) in 19 WWTPs from influent (top panel) and final effluent (bottom panel) sampling points. PFUdA, PFTrA, 6:2 Cl-PFESA and 8:2 Cl-PFESA are not plotted as all values were <LOQ.

WWTP-2 had the highest ∑_21_PFAS in influent and final effluent, with concentrations of 410 and 520 ng L^−1^, respectively. Major contributors to ∑_21_PFAS loading at WWTP-2 were PFHxS (influent 130 ng L^−1^, effluent 190 ng L^−1^) and PFOS (influent 120 ng L^−1^, effluent 130 ng L^−1^) (Fig. 1). The WWTP operator reported that approximately 45% of the inflow at WWTP-2 is attributed to baseflows, and largely a result of groundwater infiltration. Furthermore, WWTP-2 is within a highly industrialised catchment which may be causing elevated ∑_21_PFAS levels in both groundwater and influent. Final effluent from WWTP-2 is mixed with reverse osmosis (RO) reject water. The RO process is an effective long-chain PFAA treatment, removing them from effluent then partitioning them to the RO reject water [41]. Therefore, the re-introduction of RO reject water at this WWTP is likely contributing to the elevated PFAS concentrations observed in the final effluent.

#### 3.1.2 Solid matrices

PFAS were detected in all WWTP solid samples, and the mean ∑_21_PFAS in solid samples was 34 ng g^−1^ dw (median: 12 ng g^−1^ dw; range: 2.0–130 ng g^−1^ dw) (Table 2). Mean concentrations of PFAS in solids followed the trend PFOS > PFDA > PFDoA > PFOA > PFHxA > PFHxS > PFBS > PFDS>8:2 FTS > PFTeA > PFBA > PFHpA > PFHpS>6:2 FTS. The compounds PFOS, PFDoA, and PFTeA were detected in >90% of samples, while the compounds PFOA, PFDA, PFHxA, and PFTrA were detected in 70–90% of solid samples. Six of the seven compounds with detection frequencies above 70% had a carbon chain length of eight or higher. The increased partitioning of PFAS to the solid phase within WWTPs has been associated with increasing fluoroalkyl chain length [11, 51, 55]. The calculated mean partitioning coefficients from this study reflected this trend, increasing with increased fluoroalkyl chain length, and were higher in PFSAs compared to PFCAs of similar carbon chain length; except for 6:2 FTS which displayed the lowest mean partitioning coefficient, being primarily partitioned to the aqueous phase (Table S5).

The highest mean concentration found in WWTP solids was for PFOS (mean: 14; median: 4.7; range < LOD - 90 ng g^−1^ dw). The lagoon-based treatment plant sludge and AS primary sludge displayed low PFAS concentrations compared to AS secondary sludge. The process of concentrating and recycling AS secondary sludge through the treatment process, combined with the aeration/agitation provided, likely facilitates increased secondary sludge PFAS concentrations.

#### 3.1.3 Detection of the PFOS alternatives 6:2 FTS and F–53B

6:2 FTS was detected in 99% of aqueous samples (mean 7.3 ng L^−1^) and 26% of solid samples (mean 0.26 ng g^−1^ dw), At three of the larger WWTPs (-9, 330 ML day^−1^; -18, 143 ML day^−1^; and -19, 498 ML day^−1^), elevated total PFAS loading was a result of increased 6:2 FTS in influent (56, 23 and 38 ng L^−1^, respectively). 6:2 FTS has been employed in aqueous film-forming firefighting foam (AFFF) in mixtures with fluoroalkylthioamido sulfonates [56], used as a PFOS replacement in metal plating applications [57] and is a transformation intermediate in the degradation of more complex fluorotelomer-based compounds [4]. High concentrations of 6:2 FTS in WWTP effluents have been associated with AFFF use in catchments in the USA [33]. It is possible that elevated levels of 6:2 FTS observed may be associated with AFFF use or PFOS substitution in metal plating, however, it more likely indicates the presence of a range of not yet measured precursor compounds with 6:2 FTS as an intermediate degradation product.

The compound 6:2 Cl-PFESA was only detected in 4% of aqueous samples and 16% of solid samples between LOD and LOQ (Table 2). The compound 8:2 Cl-PFESA was not detected above LOD in any aqueous sample, and in 8% of solid samples between LOD and LOQ. These compounds have been demonstrated as the major components of the commercial product F–53B after purification, with a reported 6:2 Cl-PFESA content of 77.6% and 8:2 Cl-PFESA comprising an unreported percentage of the remaining fraction [19]. F–53B is used as a PFOS alternative for mist suppression in metal plating applications used in China that has recently been detected in Chinese WWTPs and the environment [19, 24, 25]. In Australia between 2006 and 2007, 99% of the directly imported PFOS was for use as a mist suppressant in metal plating which is listed as an approved, essential use [58]. The Australian metal plating industry has no need to switch to alternatives like F–53B as PFOS is still approved for use. The low F–53B concentrations detected in this study may be a result of contamination of products sourced from markets that utilise F–53B.

#### 3.1.4 Distribution within WWTPs

**Fig. 2:**
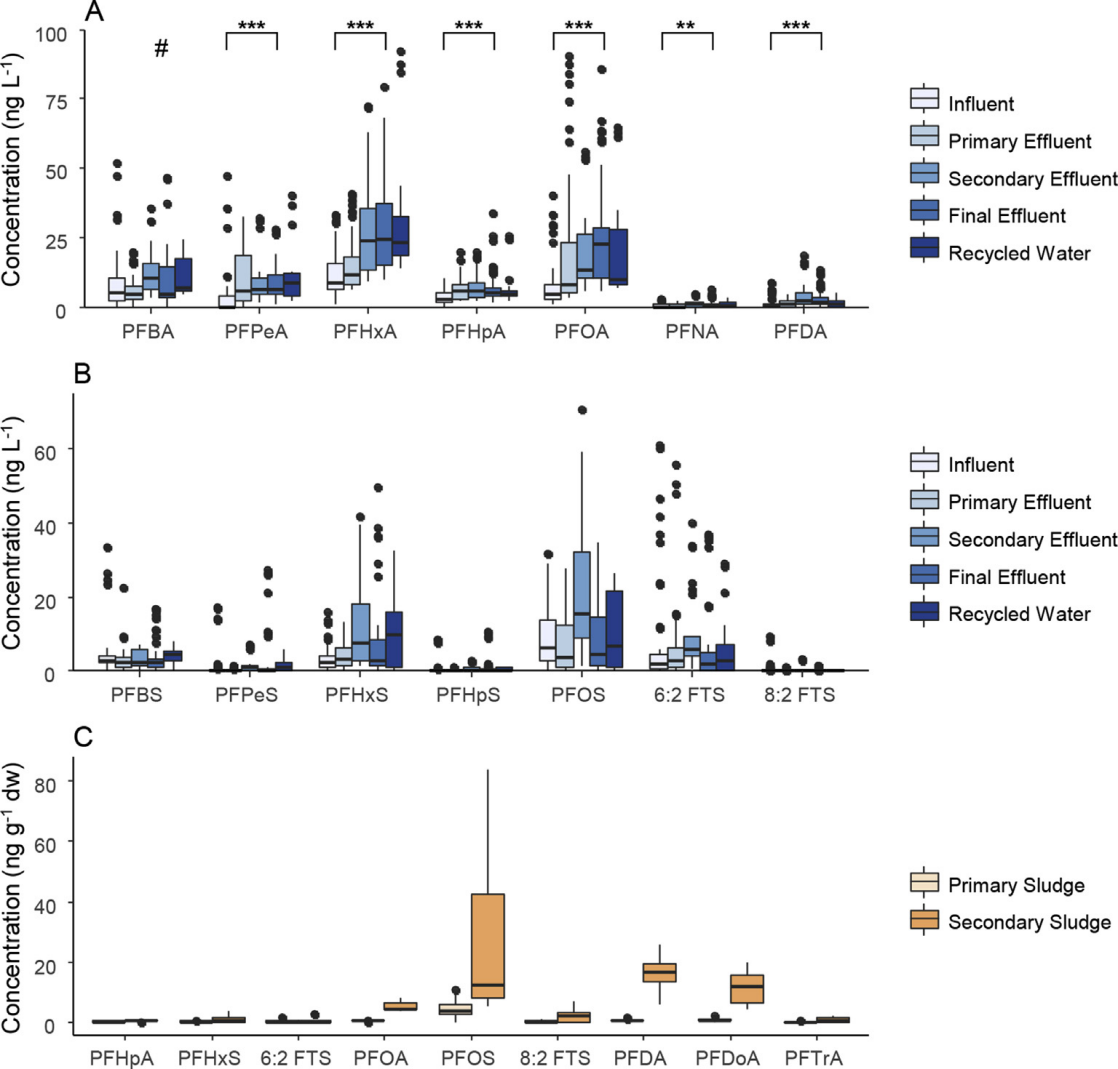
Boxplots of pooled data from 19 WWTPs for A) PFCAs (perfluorocarboxylic acids); B) PFSAs (perfluorosulfonates) and FTSs (fluorotelomer sulfonates) in aqueous samples; C) selected PFAS in solid samples. Aqueous sample locations were influent (n = 57), primary effluent (n = 39), secondary effluent (n = 24), final effluent (n = 57) and recycled water (n = 24). Solid sample locations were primary sludge (n = 15) and secondary sludge (n = 15). # indicates concentration outside y-axis range for PFBA. Asterisk (*) indicates significant difference (<0.01 = **; <0.001 = ***) between influent and final effluent concentrations when tested using linear mixed effects analysis.

PFAS concentrations generally increased in both aqueous and solid matrices through the wastewater treatment process (Fig. 2). The mean concentration of ∑_21_PFAS increased as wastewater treatment progressed from influent, to primary effluent, secondary effluent, final effluent and recycled water (76, 89, 140, 140 and 120 ng L^−1^, respectively). PFCA concentrations in aqueous samples also increased from influent to final effluent, with levels persisting in recycled water, whilst PFSA concentrations within treatment plants varied. In influent, PFOS had the highest mean concentration (17 ng L^−1^) (Table S4). PFOA had the highest mean concentration in primary effluent (23 ng L^−1^), displaying an increase from mean influent concentration (7.9 ng L^−1^). PFHxA had the highest mean concentration from pooled aqueous samples in secondary effluent, final effluent, and recycled water; increasing in concentration from influent to primary, secondary and final effluent and recycled water (11, 16, 28, 28 and 32 ng L^−1^, respectively).

Due to the delay of transmission of PFAS (caused by hydraulic retention time) within a wastewater treatment plant, the comparison of influent and effluent over the same 24-hour period may not be directly applicable. There was, however, a large variation in all PFAS concentrations between and within treatment plants from influent to final effluent. In 16 of the 19 WWTPs, ∑_21_PFAS concentrations in final effluent were greater than influent at the same WWTP, which is consistent with trends in WWTPs worldwide [11]. At WWTPs-5, 6 and 9, ∑_21_PFAS concentration was greater in influent than final effluent and largely due to PFSA and FTS concentrations.

Linear mixed-effects analysis of pooled influent and effluent data confirmed that some PFAS concentrations increased between influent and final effluent (Fig. 2). Between influent and final effluent, the compounds PFPeA, PFHxA, PFHpA, PFOA, PFNA, and PFDA (all of which are PFCAs) increased significantly. A number of transformation pathways with stable PFCA endproducts are known [18], this may explain some of the increase in PFCAs from influent to final effluent.

Microbial degradation of the compounds 6:2 PAP and 6:2 diPAP using WWTP aerobic microbes has been shown to produce 6:2 FTOH, which was then degraded further to PFHxA [59]. Furthermore, degradation of the compound 6:2 FTOH in activated sludge has been demonstrated to produce the corresponding 5:3 acid, which is then degraded further to PFHxA [34]. Little transformation of 6:2 FTOH to PFPeA was observed as the intermediary product 5:2s FTOH was likely volatilized before biotransformation could occur. This may explain the high concentrations of PFHxA compared to PFPeA (whose precursor is partitioned to the gas phase) observed in this study. PFOA has been observed as a microbial transformation product of 8:2 diPAP in soil [60] and in gilthead bream [61]. Furthermore, both PFOA and PFHxA have displayed net positive increases from influent to effluent, associated with diPAP and unknown PFAS precursor degradation in WWTP influent and sludge in three Swedish WWTPs [32]. It is likely that similar precursor transformation processes are occurring within our studied WWTPs, contributing to increased PFCA concentrations as treatment progresses.

The concentration of PFOS, PFDA, and PFDoA was higher in sludge, compared to other PFAS (Table S4). The median concentration of PFOS, PFDA, and PFDoA increased between primary and secondary sludge from 3,8 – 12, <LOQ – 17 and <LOQ – 14 ng g^−1^ dw, respectively (Fig. 2, Table S4). This increase between primary and secondary sludge was also reflected in the calculated distribution coefficients (Table S5); where coefficients increased between primary and secondary locations by 0.17–1.22 log units for PFOS and 0.37 to 1.34 log units for PFDA.

### 3.2 Trends, correlations, and transformation

Pearson correlation coefficients were positive for all PFAS measured in influent (Figure S1). In influent, positive, strong (r > 0.70) and significant (p < 0.05) correlations were displayed between compounds within the same compound class PFCAs (PFHxA-PFOA, PFHxA-PFNA, PFHxA-PFDA, and PFOA-PFDA) and between PFPeA-6:2 FTS and PFPeA-PFHxS.

In final effluent, there were significant, positive, strong correlations between PFPeA-PFHxA, PFPeA-PFOA, PFHxA-PFOA, PFHxA-PFNA, PFHpA-PFHxA, PFHpA-PFNA, PFHpA-PFHxS, PFHpA-PFOS, and PFHxS-PFOS. There was only one significant negative correlation between 6:2 FTS-PFOA (r = 0.3). There were no significant correlations for the following: 1) PFBA and all other compounds; 2) 6:2 FTS and 5 of the eleven compounds; 3) PFBS-PFOA and PFBS-PFDA; 4) PFHxS-PFPeA and PFHxS-PFOA; 5) PFOS-PFPeA, PFOS-PFOA, and PFOS-PFDA. PFCAs and PFSAs were not strongly correlated in final effluent. This implies the distribution of PFCAs and PFSAs are WWTP specific and vary in final effluent independently of each other.

**Fig. 3:**
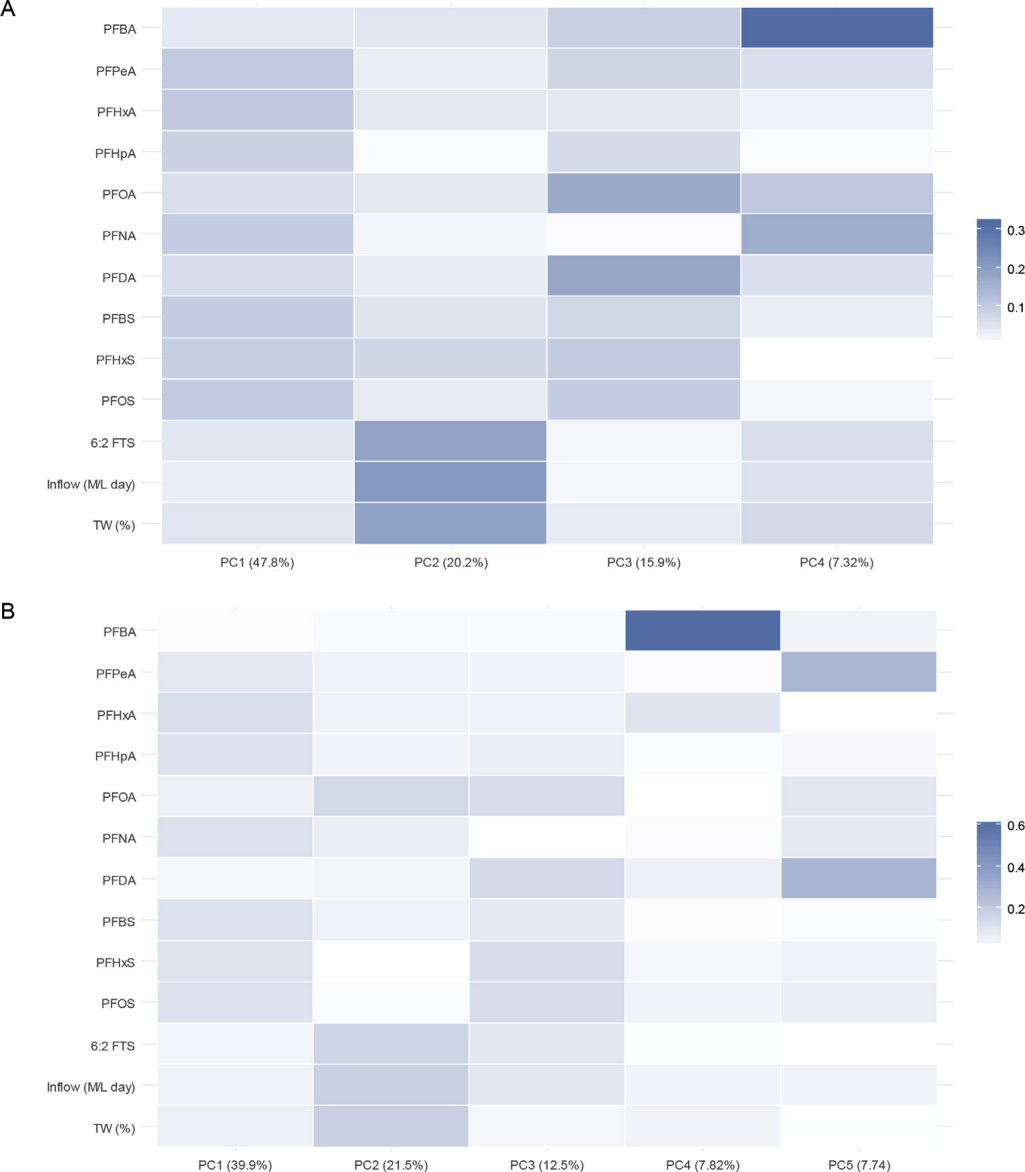
Heatmap of PCA results for principal components computed using the correlation matrix (scaled) and including average daily inflow and proportion of trade waste in inflow. Components with eigenvalues above 1 from influent (A) and final effluent (B) are displayed.

There were four principal components in the influent data, and five principal components in the final effluent data with eigenvalues above 1 (Fig. 3). For the influent data, the first four components explained 93.2% of the variation (47.8, 20.2, 15.9 and 7.32%, respectively). In component 1, the PFSAs (PFBS, PFHxS, PFOS), odd chained PFCAs (PFPeA, PFHpA, and PFNA), and PFHxA displayed strong associations and accounted for a large proportion of the variation within the data. Short-odd chain PFCAs (<C8) and PFHxA have been associated as impurities, degradants and metabolites of the short-chain fluorochemistries used to replace PFOA [4, 18, 62] Furthermore, in Australia, PFOS is still employed in approved uses and there are no current restrictions on PFHxS or PFBS [13]. The strong associations of these compounds and their contribution to the observed variation in influent data may reflect Australian PFAS usage trends and PFAS loading within specific WWTP catchments. Principal component 2 showed strong associations between WWTP daily inflow, percentage trade waste, and 6:2 FTS. This strong association was largely a result of the larger WWTPs accepting a higher proportion of trade waste, however, it shows the importance of 6:2 FTS as a possible trade waste indicator in these Australian WWTPs. In component 3, PFOA and PFDA were highly associated and in component 4, PFBA was the main contributor to the variation observed. This may indicate that these three compounds behave independently of each of in respect to PFAS loading in influent.

For the final effluent data, the five principal components accounted for 89.5% of the variation (39.9, 21.5, 12.5, 7.82 and 7.74%, respectively). In component 1 of the final effluent, there were strong associations between PFSAs (PFBS, PFHxS, PFOS), odd chained PFCAs (PFPeA, PFHpA, and PFNA), and PFHxA. In principal component 2, there was again a strong association between daily inflow volume, percentage trade waste, and 6:2 FTS (an intermediate degradant from C6 based precursors), with the addition of PFOA. There are many demonstrated transformation pathways with PFOA as the terminal end-product [63], and the significant increase of PFOA from influent to final effluent may reflect this. The strong association of PFOA with 6:2 FTS (which showed no significant change between influent and final effluent), inflow and percentage trade waste may be a result of degradation of PFOA precursors (likely as impurities from the C6 manufacture process) associated with trade waste that are not yet measured in influent at these WWTPs. In component 3, there were strong associations between PFOS, PFHxS, PFOA, and PFDA, all of which have been used extensively in the past [4]. In principle component 4, PFBA was the main contributor to variation, and behaved independently of the other PFCAs, reflecting the trend seen in influent. The compounds PFPeA and PFDA were strongly associated, accounting for a small amount of the variation in component 5.

### 3.3 Environmental discharge in final effluent

Calculation of the estimated annual discharge at a WWTP from a single sampling campaign contains a high uncertainty due to daily and seasonal variation [64]. In Australian WWTPs, temporal variation of PFAS in influent [43] and effluent [37] has been shown to be low; with observed temporal variation in influent being more likely from pulse release as opposed to seasonal factors [43]. It is, however, useful to estimate annual discharge to compare to similar Australian studies. Daily discharge rates from the 19 WWTPs in this study varied greatly, were similar to previous Australian studies, similar to studies worldwide and were influenced primarily by daily inflow (and as an extension WWTP size; Table S6).

**Fig. 4:**
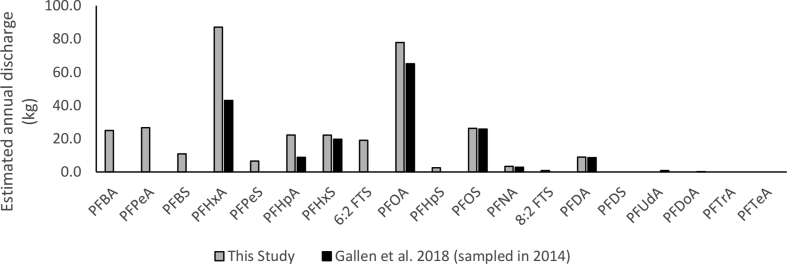
Comparison of estimated annual discharge (kg) of PFAS from Australian WWTPs in this study and from Gallen, Eaglesham [37].

PFOS and PFOA concentrations were similar to those measured in 2014 from a study of nine PFAS in effluent from in 14 Australian WWTPs [37]. In their study, they estimated a national ∑_9_PFAS discharge from Australian WWTPs as 175 kg per year in Australian WWTP effluent [37]. Assuming the same annual discharge volume of 3013 GL and using mean annual discharge rates from the 19 WWTPs in this study, we calculated an estimated discharge of ∑_21_PFAS of 339 kg. When compared, their study and our study produce similar yearly mass discharged for PFOA, PFNA, PFDA, PFHxS and PFOS (Fig. 4). In our study the estimated yearly mass discharged was higher for PFHpA (8.8 vs 22 kg annually) and PFHxA (43 vs 87 kg annually). This difference may be a result of changing PFAS use patterns or bias introduced through the WWTPs selected and sampling design each study.

The annual mass discharge of three compounds not measured in [37]: PFBA, PFPeA and 6:2 FTS (25, 27 and 19 kg annually), were similar to that of PFOS calculated for both studies (26 vs 26 kg annually). If this is the case for three compounds, and as there are now over 4700 listed PFAS in the environment [10], it is likely both studies have underestimated the total PFAS emissions from Australian WWTPs.

## 4 Conclusions

Twenty-one PFAS from four classes (PFCAs, PFSA, FTS, F–53B) were measured in aqueous and solid samples from the 19 Australian WWTPs. PFAS was detected in every sample analysed. Many PFAS were highly correlated, suggesting similar sources of PFSAs and PFCAs and independent behavior of these compound classes within WWTPs. Statistical analyses showed an increase of PFPeA, PFHxA, PFHpA, PFOA, PFNA, and PFDA between influent and final effluent. When compared to Australian WWTP PFAS emission data measured in 2014, the estimated annual discharge for the newly reported compounds PFBA, PFPeA and 6:2 FTS (25, 27 and 19 kg annually) were similar to PFOS (26 kg annually). This demonstrated that it is likely both studies have significantly underestimated the total PFAS emissions from Australian WWTPs and future work is required to determine the risk profile of PFAS present and total PFAS loading at Australian WWTPs.

The compounds 6:2 FTS and 8:2 FTS quantified, and F–53B components 6:2 Cl-PFESA and 8:2 Cl-PFESA were detected in Australian WWTPs. 6:2 FTS was strongly associated with the proportion of trade waste in influent, was partitioned to the aqueous phase, had a similar estimated Australia-wide annual mass discharged in effluent to PFOS, and did not significantly decrease between influent and final effluent. Although the ecological risk of 6:2 FTS is considered low, there are many unknowns regarding the environmental fate and effects and its presence likely indicates the degradation of currently employed short-chain fluorochemistries. In Australia, the presence of 6:2 FTS may be an emerging concern in Australian WWTPs and aqueous environments receiving WWTP effluent.

## Declarations

### Author contribution statement

Timothy Coggan: Conceived and designed the experiments; Performed the experiments; Analyzed and interpreted the data; Wrote the paper.

Damien Moodie: Performed the experiments; Contributed reagents, materials, analysis tools or data.

Adam Kolobaric, Drew Szabo: Performed the experiments.

Jeff Shimeta, Nicholas Crosbie, Elliot Lee, Milena Fernandes: Conceived and designed the experiments; Contributed reagents, materials, analysis tools or data.

Bradley Clarke: Conceived and designed the experiments.

### Funding statement

This work was supported by an Australian Government Research Training Program (RTP) Scholarship. Funding support was provided by Water Research Australia Limited, Water Corporation (WA), SA Water and Melbourne Water.

### Competing interest statement

The authors declare the following conflict of interests: Nicholas Crosbie is an employee of Melbourne Water who provided samples and funding. Elliot Lee is an employee of Water Corporation who provided samples and funding. Milena Fernandes is an employee of SA Water who provided samples and funding. Timothy Coggan, Damien Moodie and Drew Szabo have scholarship support from Melbourne Water through Water Research Australia. Bradley Clarke receives some research funding from Melbourne Water. Jeff Shimeta and Bradley Clarke are PhD supervisors for Timothy Coggan, Drew Szabo and Damien Moodie. Adam Kolobaric declares no conflicts of interest.

### Additional information

No additional information is available for this paper.

## Appendix A Supplementary data

HELIYON_2009_4157-SUPP-Rev.2 HELIYON_2009_4157-SUPP-Rev.2

## References

[1] Giesy J.P., Kannan K. (2001). Global distribution of perfluorooctane sulfonate in wildlife. Environ. Sci. Technol..

[2] Ahrens L., Bundschuh M. (2014). Fate and effects of poly- and perfluoroalkyl substances in the aquatic environment. Review.

[3] Kannan K. (2011). Perfluoroalkyl and polyfluoroalkyl substances: current and future perspectives. Environ. Chem..

[4] Buck R.C., Franklin J., Berger U., Conder J.M., Cousins I.T., de Voogt P. (2011). Perfluoroalkyl and polyfluoroalkyl substances in the environment: terminology, classification, and origins. Integr. Environ. Assess. Manag..

[5] Prevedouros K., Cousins I.T., Buck R.C., Korzeniowski S.H. (2006). Sources, fate and transport of perfluorocarboxylates. Environ. Sci. Technol..

[6] Armitage J.M., MacLeod M., Cousins I.T. (2009). Comparative assessment of the global fate and transport pathways of long-chain perfluorocarboxylic acids (PFCAs) and perfluorocarboxylates (PFCs) emitted from direct sources. Environ. Sci. Technol..

[7] Lindstrom A.B., Strynar M.J., Libelo E.L. (2011). Polyfluorinated compounds: past, present, and future. Environ. Sci. Technol..

[8] Valsecchi S., Conti D., Crebelli R., Polesello S., Rusconi M., Mazzoni M. (2016). Deriving environmental quality standards for perfluorooctanoic acid (PFOA) and related short chain perfluorinated alkyl acids. J. Hazard Mater..

[9] Wang Z., DeWitt J.C., Higgins C.P., Cousins I.T. (2017). A never-ending story of per- and polyfluoroalkyl substances (PFASs)?. Environ. Sci. Technol..

[10] OECD (2018). Toward A New Comprehensive Global Database of Per - and Polyfluoroalkyl Substances (PFASs): Summary Report on Updating the OECD 2007 List of PER- and Polyfluoroalkyl Substances (PFASs). Paris.

[11] Arvaniti O.S., Stasinakis A.S. (2015). Review on the occurrence, fate and removal of perfluorinated compounds during wastewater treatment. Sci. Total Environ..

[12] Hu X.C., Andrews D.Q., Lindstrom A.B., Bruton T.A., Schaider L.A., Grandjean P. (2016). Detection of poly- and perfluoroalkyl substances (PFASs) in U.S. Drinking water linked to industrial sites, military fire training areas, and wastewater treatment plants. Environ. Sci. Technol. Lett..

[13] (2018). HEPA HotEAaNZ. PFAS National Environmental Management Plan. Australia.

[14] Giesy J.P., Naile J.E., Khim J.S., Jones P.D., Newsted J.L. (2010). Aquatic toxicology of perfluorinated chemicals. Reviews of environmental contamination and toxicology. Rev. Environ. Contam. Toxicol..

[15] Toms L.-M.L., Calafat A.M., Kato K., Thompson J., Harden F., Hobson P. (2009). Polyfluoroalkyl chemicals in pooled blood serum from infants, children, and adults in Australia. Environ. Sci. Technol..

[16] Higgins C.P., Luthy R.G. (2006). Sorption of perfluorinated surfactants on sediments. Environ. Sci. Technol..

[17] Zareitalabad P., Siemens J., Hamer M., Amelung W. (2013). Perfluorooctanoic acid (PFOA) and perfluorooctanesulfonic acid (PFOS) in surface waters, sediments, soils and wastewater – a review on concentrations and distribution coefficients. Chemosphere.

[18] Liu J., Mejia Avendaño S. (2013). Microbial degradation of polyfluoroalkyl chemicals in the environment: a review. Environ. Int..

[19] Ruan T., Lin Y., Wang T., Liu R., Jiang G. (2015). Identification of novel polyfluorinated ether sulfonates as PFOS alternatives in municipal sewage sludge in China. Environ. Sci. Technol..

[20] Xiao F. (2017). Emerging poly- and perfluoroalkyl substances in the aquatic environment: a review of current literature. Water Res..

[21] UNEP (2012). Technical Paper on the Identification and Assessment of Alternatives to the Use of Perfluorooctane Sulfonic Acid in Open Applications (UNEP/POPS/POPRC.8/INF/17).

[22] Hoke R.A., Ferrell B.D., Ryan T., Sloman T.L., Green J.W., Nabb D.L. (2015). Aquatic hazard, bioaccumulation and screening risk assessment for 6:2 fluorotelomer sulfonate. Chemosphere.

[23] Shi G., Cui Q., Pan Y., Sheng N., Sun S., Guo Y. (2017). 6:2 Chlorinated polyfluorinated ether sulfonate, a PFOS alternative, induces embryotoxicity and disrupts cardiac development in zebrafish embryos. Aquat. Toxicol..

[24] Wang S., Huang J., Yang Y., Hui Y., Ge Y., Larssen T. (2013). First report of a Chinese PFOS alternative overlooked for 30 Years: its toxicity, persistence, and presence in the environment. Environ. Sci. Technol..

[25] Shi Y., Vestergren R., Zhou Z., Song X., Xu L., Liang Y. (2015). Tissue distribution and whole body burden of the chlorinated polyfluoroalkyl ether sulfonic acid F-53B in crucian carp (Carassius carassius): evidence for a highly bioaccumulative contaminant of emerging concern. Environ. Sci. Technol..

[26] Becker A.M., Gerstmann S., Frank H. (2008). Perfluorooctane surfactants in waste waters, the major source of river pollution. Chemosphere.

[27] Arvaniti O.S., Andersen H.R., Thomaidis N.S., Stasinakis A.S. (2014). Sorption of Perfluorinated Compounds onto different types of sewage sludge and assessment of its importance during wastewater treatment. Chemosphere.

[28] Sun H., Gerecke A.C., Giger W., Alder A.C. (2011). Long-chain perfluorinated chemicals in digested sewage sludges in Switzerland. Environ. Pollut..

[29] Zhou Q., Deng S., Zhang Q., Fan Q., Huang J., Yu G. (2010). Sorption of perfluorooctane sulfonate and perfluorooctanoate on activated sludge. Chemosphere.

[30] Zhang C., Yan H., Li F., Hu X., Zhou Q. (2013). Sorption of short- and long-chain perfluoroalkyl surfactants on sewage sludges. J. Hazard Mater..

[31] Filipovic M., Berger U. (2015). Are perfluoroalkyl acids in waste water treatment plant effluents the result of primary emissions from the technosphere or of environmental recirculation?. Chemosphere.

[32] Eriksson U., Haglund P., Kärrman A. (2017). Contribution of precursor compounds to the release of per- and polyfluoroalkyl substances (PFASs) from waste water treatment plants (WWTPs). J. Environ. Sci..

[33] Houtz E.F., Sutton R., Park J.-S., Sedlak M. (2016). Poly- and perfluoroalkyl substances in wastewater: significance of unknown precursors, manufacturing shifts, and likely AFFF impacts. Water Res..

[34] Zhao L., McCausland P.K., Folsom P.W., Wolstenholme B.W., Sun H., Wang N. (2013). 6:2 Fluorotelomer alcohol aerobic biotransformation in activated sludge from two domestic wastewater treatment plants. Chemosphere.

[35] Wang N., Liu J., Buck R.C., Korzeniowski S.H., Wolstenholme B.W., Folsom P.W. (2011). 6:2 Fluorotelomer sulfonate aerobic biotransformation in activated sludge of waste water treatment plants. Chemosphere.

[36] Taylor M.D. (2018). First reports of per- and poly-fluoroalkyl substances (PFASs) in Australian native and introduced freshwater fish and crustaceans. Mar. Freshw. Res..

[37] Gallen C., Eaglesham G., Drage D., Hue N.T., Mueller J.F. (2018). A mass estimate of perfluoroalkyl substance (PFAS) release from Australian wastewater treatment plants. Chemosphere.

[38] Thompson J., Roach A., Eaglesham G., Bartkow M.E., Edge K., Mueller J.F. (2011). Perfluorinated alkyl acids in water, sediment and wildlife from Sydney Harbour and surroundings. Mar. Pollut. Bull..

[39] Baduel C., Paxman C.J., Mueller J.F. (2015). Perfluoroalkyl substances in a firefighting training ground (FTG), distribution and potential future release. J. Hazard Mater..

[40] Szabo D., Coggan T.L., Robson T.C., Currell M., Clarke B.O. (2018). Investigating recycled water use as a diffuse source of per- and polyfluoroalkyl substances (PFASs) to groundwater in Melbourne, Australia. Sci. Total Environ..

[41] Thompson J., Eaglesham G., Reungoat J., Poussade Y., Bartkow M., Lawrence M. (2011). Removal of PFOS, PFOA and other perfluoroalkyl acids at water reclamation plants in South East Queensland Australia. Chemosphere.

[42] Blackbeard J., Lloyd J., Magyar M., Mieog J., Linden K.G., Lester Y. (2016). Demonstrating organic contaminant removal in an ozone-based water reuse process at full scale. Environ. Sci.: Water Res. Technol..

[43] Nguyen H.T., Kaserzon S.L., Thai P.K., Vijayasarathy S., Bräunig J., Crosbie N.D. (2019). Temporal trends of per- and polyfluoroalkyl substances (PFAS) in the influent of two of the largest wastewater treatment plants in Australia. Emerg. Contam..

[44] NICNAS (2013). Perfluorinated Chemicals (PFCs) Factsheet.

[45] Hepburn E., Madden C., Szabo D., Coggan T.L., Clarke B., Currell M. (2019). Contamination of groundwater with per- and polyfluoroalkyl substances (PFAS) from legacy landfills in an urban re-development precinct. Environ. Pollut..

[46] Coggan T.L., Anumol T., Pyke J., Shimeta J., Clarke B.O. (2019). A single analytical method for the determination of 53 legacy and emerging per- and polyfluoroalkyl substances (PFAS) in aqueous matrices. Anal. Bioanal. Chem..

[47] Gallen C., Drage D., Kaserzon S., Baduel C., Gallen M., Banks A. (2016). Occurrence and distribution of brominated flame retardants and perfluoroalkyl substances in Australian landfill leachate and biosolids. J. Hazard Mater..

[48] R Core Team (2017). R: A Language and Environment for Statistical Computing.

[49] Wickham H. (2007). Reshaping Data with the reshape package. J. Stat. Softw..

[50] Wickam H. (2009). ggplot2: Elegant Graphics for Data Analysis. http://ggplot2.org.

[51] Sun H., Zhang X., Wang L., Zhang T., Li F., He N. (2012). Perfluoroalkyl compounds in municipal WWTPs in Tianjin, China—concentrations, distribution and mass flow. Environ. Sci. Pollut. Control Ser..

[52] Bates D., Maechler M., Bolker B., Walker S. (2015). Fitting linear mixed-effects models using lme4. J. Stat. Softw..

[53] Kassambara A., Mundt F. (2017). Factoextra: Extract and Visualize the Results of Multivariate Data Analyses R Package Version 1.0.5. https://CRAN.R-project.org/package=factoextra.

[54] Kotthoff M., Bücking M. (2018). Four chemical trends will shape the next decade's directions in perfluoroalkyl and polyfluoroalkyl substances research. Front. Chem..

[55] Eriksson U., Kaerrman A. (2015). World-wide indoor exposure to polyfluoroalkyl phosphate esters (PAPs) and other PFASs in household dust. Environ. Sci. Technol..

[56] Schultz M.M., Barofsky D.F., Field J.A. (2004). Quantitative determination of fluorotelomer sulfonates in groundwater by LC MS/MS. Environ. Sci. Technol..

[57] Wang Z., Cousins I.T., Scheringer M., Hungerbühler K. (2013). Fluorinated alternatives to long-chain perfluoroalkyl carboxylic acids (PFCAs), perfluoroalkane sulfonic acids (PFSAs) and their potential precursors. Environ. Int..

[58] NICNAS. Alert (2008). Perfluorooctane Sulfonate (PFOS) and Perfluoroalkyl Sulfonate (PFAS). https://www.nicnas.gov.au/chemical-information/Topics-of-interest2/subjects/Per-and-poly-fluorinated-alkyl-substances/pfc-derivatives-and-chemicals-on-which-they-are-based.

[59] Lee H., D'Eon J., Mabury S.A. (2010). Biodegradation of polyfluoroalkyl phosphates as a source of perfluorinated acids to the environment. Environ. Sci. Technol..

[60] Liu C., Liu J. (2016). Aerobic biotransformation of polyfluoroalkyl phosphate esters (PAPs) in soil. Environ. Pollut..

[61] Zabaleta I., Bizkarguenaga E., Izagirre U., Negreira N., Covaci A., Benskin J.P. (2017). Biotransformation of 8:2 polyfluoroalkyl phosphate diester in gilthead bream (Sparus aurata). Sci. Total Environ..

[62] Luz A.L., Anderson J.K., Goodrum P., Durda J. (2019). Perfluorohexanoic acid toxicity, part I: development of a chronic human health toxicity value for use in risk assessment. Regul. Toxicol. Pharmacol..

[63] Ruan T., Lin Y., Wang T., Jiang G., Wang N. (2015). Methodology for studying biotransformation of polyfluoroalkyl precursors in the environment. Trac. Trends Anal. Chem..

[64] Department of Environment and Energy (2011). National Pollutant Inventory Emission Estimation Technique Manual for Sewage and Wastewater Treatment.

