# Supplementary material for: An investigation into per- and polyfluoroalkyl substances (PFAS) in nineteen Australian wastewater treatment plants (WWTPs)
Source: Heliyon. 2019 Aug 23;5(8):e02316. doi: 10.1016/j.heliyon.2019.e02316 (PMC6716228; doi:10.1016/j.heliyon.2019.e02316)
Supplement: HELIYON_2009_4157-SUPP-Rev.2 [file mmc1.docx]

An Investigation into Per- and Polyfluoroalkyl Substances (PFAS) in Nineteen Australian Wastewater Treatment Plants (WWTPs)

Timothy L. Coggan^1^*, Damien Moodie^1^, Adam Kolobaric^1^, Drew Szabo^1^, Jeff Shimeta^1^, Nicholas D. Crosbie^2,3^, Elliot Lee^4^, Milena Fernandes^5, 6^ & Bradley O. Clarke^1^

^1^ Centre for Environmental Sustainability and Remediation, School of Science, RMIT University, GPO Box 2476, Melbourne, Vic. 3001, Australia

^2^ Applied Research, Melbourne Water Corporation, Docklands, VIC 3001, Australia

^3^ Faculty of Engineering, University of New South Wales, NSW 2052, Australia

^4^ Water Corporation, Leederville, Western Australia, 6007, Australia

^5^ SA Water, GPO Box 1751, Adelaide SA 5001, Australia

^6^ College of Science and Engineering, Flinders University, Adelaide, Australia

*Corresponding author

[timothy.coggan@rmit.edu.au](mailto:timothy.coggan@rmit.edu.au)

# Supplementary Information

The analysis was performed using liquid chromatography-tandem mass spectrometry (LC-MS/MS) on an Agilent 6495B mass spectrometer coupled with an Agilent 1290 II Infinity liquid chromatograph optimised for PFAS analysis. Twenty-one PFAS compounds were quantified using isotope dilution listed in Table S1 with mass-labelled surrogate employed.

| Table S1. Compound names, chemical identifier, number of carbons in compound (C), and MS/MS parameters for analytical method used in this study. | | | | | | | | |
| --- | --- | --- | --- | --- | --- | --- | --- | --- |
| **Compound name** | **C** | **Acronym** | **CAS No** | **Prec (m/z)** | **Prod (m/z)** | **CE (V)** | **Rt (min)** | **Surrogate** |
| Perfluorotetradecanoic acid | 14 | PFTeA | 376-06-7 | 712.9 | 668.9(168.9) | 10(38) | 9.45 | PFTeA-13C2 |
| Perfluorotridecanoic acid | 13 | PFTrA | 72629-94-8 | 663 | 618.9(168.9) | 14(34) | 8.945 | PFTeA-13C2 |
| Perfluorododecanoic acid | 12 | PFDoA | 307-55-1 | 612.9 | 569(319) | 14(22) | 8.48 | PFDoA-13C2 |
| Perfluoroundecanoic acid | 11 | PFUnA | 2058-94-8 | 563 | 518.9(268.9) | 12(16) | 7.955 | PFDA-13C2 |
| Perfluorodecanoic acid | 10 | PFDA | 335-76-2 | 512.9 | 469(268.9) | 6(18) | 7.371 | PFDA-13C2 |
| Perfluorononanoic acid | 9 | PFNA | 375-95-1 | 463 | 418.9(218.9) | 10(18) | 6.717 | PFDA-13C2 |
| Perfluorooctanoic acid | 8 | PFOA | 335-67-1 | 413 | 368.9(169) | 6(18) | 6.024 | PFOA-13C8 |
| Perfluoroheptanoic acid | 7 | PFHpA | 375-85-9 | 363 | 318.9(168.9) | 6(18) | 5.358 | PFOA-13C8 |
| Perfluorohexanoic acid | 6 | PFHxA | 307-24-4 | 313 | 269(119) | 6(22) | 4.759 | PFHxA-13C2 |
| Perfluoropentanoic acid | 5 | PFPeA | 2706-90-3 | 263 | 219 | 6 | 4.202 | PFPeA-13C3 |
| Perfluorobutanoic acid | 4 | PFBA | 375-22-4 | 213 | 169 | 6 | 2.64 | PFBA-13C3 |
| Perfluorodecane sulfonic acid | 10 | PFDS | 335-77-3 | 598.9 | 80(98.9) | 60(60) | 7.941 | PFOS-13C4 |
| Perfluorooctane sulfonic acid | 8 | PFOS | 1763-23-1 | 498.9 | 80(99) | 56(56) | 6.734 | PFOS-13C4 |
| Perfluoroheptane sulfonic acid | 7 | PFHpS | 375-92-8 | 449 | 80(99) | 50(46) | 6.059 | PFOS-13C4 |
| Perfluorohexane sulfonic acid | 6 | PFHxS | 355-46-4 | 399 | 80(99, 119) | 48(44, 44) | 5.414 | PFHxS-13C3 |
| Perfluoropentane sulfonic acid | 5 | PFPeS | 2706-91-4 | 348.9 | 80(99) | 40(36) | 4.84 | PFHxS-13C3 |
| Perfluorobutane sulfonic acid | 4 | PFBS | 375-73-5 | 299 | 80(99) | 44(36) | 4.347 | PFBS-13C2 |
| 6:2 Fluorotelomer sulfonic acid | 8 (6 CF) | 6:2 FTS | 27619-97-2 | 426.9 | 407(81, 80) | 28(44, 44) | 5.994 | 6:2 FTS-13C |
| 8:2 Fluorotelomer sulfonic acid | 10 (8 CF) | 8:2 FTS | 39108-34-4 | 526.9 | 507(80) | 32(52) | 7.351 | 6:2 FTS-13C |
| 6:2 Chlorinated perfluoroether sulfonic acid (F-53B) | 8 (6 CF) | 6:2 Cl-PFESA | 73606-19-6 | 530.9 | 350.9(98.9, 83) | 28(28, 32) | 7.114 | PFOS-13C4 |
| 8:2 Chlorinated perfluoroether sulfonic acid | 10 (8 CF) | 8:2 Cl-PFESA | 83329-89-9 | 630.9 | 451(98.9, 83) | 32(32, 42) | 8.257 | PFOS-13C4 |

For quality control, aqueous samples were extracted in batches containing two method blanks and a laboratory control sample (LCS). Solid LCS samples consisted of acid-washed sand spiked with 10 ng of PFAS and extracted alongside batches. Results are listed in Table S2.

| Table S2. Mean LCS recovery and standard deviation from extracted aqueous batches for spikes of 5 ng (n=4), 1 ng (n=1) and 0.25 ng (n=2) and solid batches (n=3). | | | | |
| --- | --- | --- | --- | --- |
|  | **LCS-Aqueous** |  | **LCS-Solids** |  |
| **Compound** | **Mean recovery (%)** | **s.d.** | **Mean recovery (%)** | **s.d.** |
| **PFBA** | 90% | 8% | 102% | 6% |
| **PFPeA** | 94% | 9% | 97% | 6% |
| **PFBS** | 104% | 11% | 117% | 2% |
| **PFHxA** | 103% | 8% | 97% | 2% |
| **PFPeS** | 114% | 10% | 88% | 3% |
| **PFHpA** | 94% | 10% | 94% | 5% |
| **PFHxS** | 102% | 11% | 93% | 5% |
| **6:2 FTS** | 99% | 13% | 61% | 8% |
| **PFOA** | 90% | 5% | 95% | 2% |
| **PFHpS** | 112% | 12% | 98% | 5% |
| **PFNA** | 90% | 11% | 84% | 4% |
| **PFOS** | 91% | 6% | 96% | 5% |
| **6:2 Cl-PFAES** | 89% | 9% | 97% | 6% |
| **8:2 FTS** | 99% | 14% | 102% | 10% |
| **PFDA** | 93% | 5% | 99% | 7% |
| **PFDS** | 72% | 13% | 98% | 8% |
| **PFUdA** | 93% | 10% | 117% | 6% |
| **8:2 Cl-PFAES** | 73% | 7% | 103% | 6% |
| **PFDoA** | 81% | 5% | 103% | 2% |
| **PFTrA** | 70% | 6% | 110% | 2% |
| **PFTeA** | 76% | 15% | 104% | 6% |

Aqueous samples (influent, primary effluent, secondary effluent, final effluent, recycled water) consisting of either triplicate sub-samples from a single 24 hr composite or three replicate grab samples were collected in 250 mL polypropylene bottles pre-rinsed with ultrapure water, methanol, and site water. Solid samples (primary sludge, secondary sludge, lagoon sludge, and one lagoon sludge dredge pile) were collected in 50 mL polypropylene centrifuge tubes. Summary of results are listed in Table S3 and S4.

| Table S3. Summary of PFAS concentrations (ng L^-1^) at each aqueous sample point. Compounds marked with and asterisk (*) indicate that the sum branched plus linear are reported. | | | | | | | | | | | | | | | | | | | | | | |
| --- | --- | --- | --- | --- | --- | --- | --- | --- | --- | --- | --- | --- | --- | --- | --- | --- | --- | --- | --- | --- | --- | --- |
|  | **PFBA** | **PFPeA** | **PFHxA** | **PFHpA** | **PFOA** | **PFNA** | **PFDA** | **PFUdA** | **PFDoA** | **PFTrA** | **PFTeA** | **PFBS** | **PFPeS*** | **PFHxS*** | **PFHpS*** | **PFOS*** | **PFDS** | **6:2 FTS** | **8:2 FTS** | **6:2 Cl-PFAES** | **8:2 Cl-PFAES** | **SUM 21 PFAS** |
| **Influent (n=64)** |  |  |  |  |  |  |  |  |  |  |  |  |  |  |  |  |  |  |  |  |  |  |
| Median (ng/L) | 5.25 | <LOQ | **8.65** | 2.80 | 4.35 | <LOQ | 0.60 | <LOD | <LOQ | <LOD | <LOD | 2.55 | <LOQ | 2.30 | <LOQ | 6.95 | <LOD | 1.70 | <LOQ | <LOD | <LOD | 45.2 |
| Mean (ng/L) | 8.12 | 4.75 | 11.4 | 3.60 | 7.92 | 0.67 | 1.42 | <LOQ | 0.61 | <LOQ | 0.36 | 4.73 | 1.76 | 12.6 | 0.92 | 17.0 | 0.30 | 7.29 | 0.84 | <LOQ | <LOD | 75.7 |
| s.d (ng/L) | 10.1 | 9.12 | 8.28 | 2.61 | 8.91 | 0.70 | 2.07 |  | 0.59 |  | 0.29 | 7.09 | 3.74 | 32.2 | 1.98 | 27.8 | 0.21 | 14.1 | 1.83 |  |  |  |
| min (ng/L) | <LOQ | <LOD | 1.40 | <LOQ | 1.00 | <LOD | <LOD | <LOD | <LOD | <LOD | <LOD | <LOQ | <LOD | <LOD | <LOD | <LOD | <LOD | <LOQ | <LOD | <LOD | <LOD | 9.27 |
| max (ng/L) | 52.1 | 47.3 | 33.5 | 10.4 | 40.5 | 2.60 | 9.00 | <LOQ | 2.20 | <LOQ | 2.00 | 33.3 | 17.0 | 142 | 8.60 | 129 | 1.10 | 61.1 | 9.20 | <LOQ | <LOD | 412 |
| Detect (%) | 100% | 86% | 100% | 100% | 100% | 91% | 94% | 5% | 73% | 22% | 36% | 100% | 70% | 91% | 77% | 97% | 33% | 100% | 84% | 5% | 0% |  |
| **Primary effluent (n=39)** |  |  |  |  |  |  |  |  |  |  |  |  |  |  |  |  |  |  |  |  |  |  |
| Median (ng/L) | 4.60 | 5.6 | **11.9** | 5.60 | 8.10 | <LOQ | 1.40 | <LOD | <LOD | <LOD | <LOD | 2.40 | 0.20 | 3.30 | <LOQ | 3.80 | <LOD | 2.70 | <LOQ | <LOD | <LOD | 76.0 |
| Mean (ng/L) | 6.19 | 10.1 | 16.2 | 6.43 | 23.1 | 0.71 | 1.56 | <LOQ | 0.80 | <LOQ | 0.27 | 3.13 | 0.37 | 4.43 | <LOQ | 8.70 | <LOQ | 7.72 | <LOQ | <LOQ | <LOD | 89.4 |
| s.d (ng/L) | 5.43 | 9.47 | 11.0 | 3.96 | 28.0 | 0.66 | 1.33 |  | 0.97 |  | 0.15 | 3.80 | 0.31 | 3.49 |  | 9.56 |  | 13.4 |  |  |  |  |
| min (ng/L) | <LOQ | <LOQ | 6.30 | 2.10 | 3.20 | <LOQ | <LOQ | <LOD | <LOD | <LOD | <LOD | <LOQ | <LOD | <LOD | <LOD | <LOQ | <LOD | <LOQ | <LOD | <LOD | <LOD | 36.5 |
| max (ng/L) | 19.8 | 32.5 | 41.1 | 19.7 | 90.7 | 2.50 | 4.40 | <LOQ | 4.20 | <LOQ | 0.60 | 22.6 | 1.30 | 13.1 | 0.90 | 27.7 | <LOQ | 55.7 | 0.80 | <LOQ | <LOD | 180 |
| Detect (%) | 100% | 100% | 100% | 100% | 100% | 100% | 100% | 8% | 44% | 18% | 23% | 100% | 56% | 97% | 69% | 100% | 10% | 100% | 87% | 8% | 0% |  |
| **Secondary effluent (n=26)** |  |  |  |  |  |  |  |  |  |  |  |  |  |  |  |  |  |  |  |  |  |  |
| Median (ng/L) | 10.6 | 6.15 | **23.7** | 5.55 | 13.2 | 1.10 | 2.35 | <LOD | <LOQ | <LOD | <LOD | 2.5 | 0.80 | 7.7 | <LOQ | 15.65 | <LOD | 5.95 | <LOQ | <LOD | <LOD | 141 |
| Mean (ng/L) | 13.0 | 9.44 | 27.6 | 7.76 | 20.2 | 1.29 | 4.49 | <LOQ | <LOQ | <LOQ | <LOQ | 3.41 | 1.45 | 13.0 | 0.84 | 24.0 | <LOQ | 10.2 | 0.68 | <LOQ | <LOD | 142 |
| s.d (ng/L) | 8.73 | 8.37 | 17.8 | 5.35 | 14.7 | 1.32 | 5.00 |  |  |  |  | 2.37 | 1.96 | 12.6 | 0.86 | 20.2 |  | 11.2 | 0.95 |  |  |  |
| min (ng/L) | 3.50 | 1.90 | 9.20 | 2.50 | 6.00 | <LOQ | <LOQ | <LOD | <LOD | <LOD | <LOD | <LOQ | <LOQ | 1.60 | <LOD | 1.60 | <LOD | 0.60 | <LOD | <LOD | <LOD | 50.8 |
| max (ng/L) | 35.3 | 32.3 | 72.1 | 20.0 | 56.0 | 4.90 | 18.4 | <LOQ | <LOQ | <LOQ | 0.80 | 7.30 | 7.00 | 41.8 | 2.60 | 70.4 | <LOQ | 39.8 | 3.20 | <LOQ | <LOD | 266 |
| Detect (%) | 100% | 100% | 100% | 100% | 100% | 100% | 100% | 15% | 58% | 19% | 27% | 100% | 100% | 100% | 96% | 100% | 27% | 100% | 69% | 12% | 0% |  |
| **Final effluent (n=62)** |  |  |  |  |  |  |  |  |  |  |  |  |  |  |  |  |  |  |  |  |  |  |
| Median (ng/L) | 5.80 | 6.65 | **24.6** | 5.50 | 22.8 | 0.70 | 2.00 | <LOD | <LOD | <LOD | <LOD | 2.10 | <LOQ | 3.05 | <LOQ | 4.35 | <LOD | 1.95 | <LOQ | <LOD | <LOD | 91.1 |
| Mean (ng/L) | 22.0 | 8.68 | 28.4 | 7.23 | 24.9 | 1.11 | 2.91 | <LOQ | <LOQ | <LOQ | <LOQ | 3.79 | 2.82 | 19.9 | 1.22 | 16.0 | <LOQ | 6.13 | 0.33 | <LOD | <LOD | 142 |
| s.d (ng/L) | 59.0 | 6.12 | 16.5 | 5.75 | 18.0 | 1.34 | 3.00 |  |  |  |  | 4.30 | 6.39 | 46.8 | 2.37 | 30.2 |  | 10.4 |  |  |  |  |
| min (ng/L) | <LOQ | 1.10 | 10.0 | 1.70 | 5.90 | <LOQ | <LOD | <LOD | <LOD | <LOD | <LOD | <LOD | <LOD | <LOD | <LOD | <LOQ | <LOD | <LOD | <LOD | <LOD | <LOD | 34.1 |
| max (ng/L) | 369 | 27.8 | 79.2 | 34.1 | 85.7 | 6.60 | 13.2 | <LOQ | <LOQ | <LOQ | <LOQ | 16.9 | 27.1 | 196 | 10.7 | 141 | <LOQ | 37.0 | 1.60 | <LOD | <LOD | 517 |
| Detect (%) | 100% | 100% | 100% | 100% | 100% | 100% | 98% | 5% | 23% | 11% | 8% | 97% | 85% | 95% | 77% | 100% | 13% | 98% | 77% | 0% | 0% |  |
| **Recycled water (24)** |  |  |  |  |  |  |  |  |  |  |  |  |  |  |  |  |  |  |  |  |  |  |
| Median (ng/L) | 7.20 | 8.75 | **23.2** | 4.85 | 9.95 | <LOQ | 1.35 | <LOQ | <LOQ | <LOD | <LOD | 4.35 | 1.05 | 9.75 | <LOQ | 6.60 | <LOD | 2.70 | <LOD | <LOD | <LOD | 79.1 |
| Mean (ng/L) | 11.0 | 11.1 | 31.9 | 7.33 | 20.5 | 0.99 | 1.58 | <LOQ | <LOQ | <LOQ | <LOQ | 4.46 | 1.91 | 11.0 | 0.61 | 10.4 | <LOQ | 6.69 | <LOQ | <LOD | <LOD | 118 |
| s.d (ng/L) | 7.03 | 10.2 | 23.2 | 6.95 | 18.7 | 1.16 | 1.53 |  |  |  |  | 2.50 | 1.83 | 10.5 | 0.46 | 9.99 |  | 8.64 |  |  |  |  |
| min (ng/L) | 4.50 | 2.30 | 13.8 | 2.20 | 7.00 | <LOQ | <LOQ | <LOD | <LOD | <LOD | <LOD | <LOD | <LOD | <LOQ | <LOD | <LOQ | <LOD | <LOD | <LOD | <LOD | <LOD | 51.8 |
| max (ng/L) | 24.6 | 40.3 | 91.9 | 25.7 | 64.5 | 3.60 | 5.00 | <LOQ | <LOQ | <LOQ | <LOQ | 7.90 | 6.00 | 32.4 | 1.10 | 26.4 | <LOQ | 28.9 | <LOQ | <LOD | <LOD | 310 |
| Detect (%) | 100% | 100% | 100% | 100% | 100% | 100% | 100% | 50% | 50% | 33% | 38% | 92% | 83% | 100% | 63% | 100% | 25% | 92% | 33% | 0% | 0% |  |
|  |  |  |  |  |  |  |  |  |  |  |  |  |  |  |  |  |  |  |  |  |  |  |

| Table S4. Summary of PFAS concentrations (ng g^-1^ dw) at each solid sample point. Compounds marked with and asterisk (*) indicate that the sum branched plus linear are reported. | | | | | | | | | | | | | | | | | | | | | | |
| --- | --- | --- | --- | --- | --- | --- | --- | --- | --- | --- | --- | --- | --- | --- | --- | --- | --- | --- | --- | --- | --- | --- |
|  | **PFBA** | **PFPeA** | **PFHxA** | **PFHpA** | **PFOA** | **PFNA** | **PFDA** | **PFUdA** | **PFDoA** | **PFTrA** | **PFTeA** | **PFBS** | **PFPeS*** | **PFHxS*** | **PFHpS*** | **PFOS*** | **PFDS** | **6:2 FTS** | **8:2 FTS** | **6:2 Cl-PFAES** | **8:2 Cl-PFAES** | **SUM 21 PFAS** |
| **Primary Sludge (n=14)** |  |  |  |  |  |  |  |  |  |  |  |  |  |  |  |  |  |  |  |  |  |  |
| Median (ng/g dw) |  |  | 0.56 | <LOD | <LOQ | <LOD | <LOQ | <LOD | <LOQ | <LOD | <LOQ | <LOD | <LOD | <LOD |  | 3.78 | <LOD | <LOD | <LOD |  | <LOD | 9.62 |
| Mean (ng/g dw) |  |  | 0.74 | <LOQ | 0.29 | <LOQ | 0.42 | <LOQ | 0.59 | <LOQ | 0.22 | 0.86 | <LOQ | 0.10 |  | 4.41 | <LOQ | 0.28 | 0.23 |  | <LOQ | 8.86 |
| s.d (ng/g dw) |  |  | 0.66 |  | 0.13 |  | 0.44 |  | 0.66 |  | 0.11 | 1.92 |  | 0.19 |  | 2.97 |  | 0.62 | 0.36 |  |  |  |
| min (ng/g dw) |  |  | <LOD | <LOD | <LOD | <LOD | <LOD | <LOD | 0.1 | <LOD | <LOD | <LOD | <LOD | <LOD |  | <LOQ | <LOD | <LOD | <LOD |  | <LOD | 1.15 |
| max (ng/g dw) |  |  | 1.74 | <LOQ | 0.55 | <LOQ | 1.29 | <LOQ | 1.97 | <LOQ | 0.46 | 5.78 | <LOQ | 0.66 |  | 10.5 | 0.34 | 1.73 | 1.12 |  | <LOQ | 13.6 |
| Detect (%) | 0% | 0% | 79% | 43% | 93% | 21% | 79% | 21% | 100% | 43% | 93% | 43% | 7% | 29% | 0% | 100% | 14% | 29% | 36% | 0% | 7% |  |
|  |  |  |  |  |  |  |  |  |  |  |  |  |  |  |  |  |  |  |  |  |  |  |
| **Secondary Sludge (n=15)** | |  |  |  |  |  |  |  |  |  |  |  |  |  |  |  |  |  |  |  |  |  |
| Median (ng/g dw) |  | <LOD | 3.43 | <LOQ | 4.84 | 0.34 | 17.1 | 0.35 | 13.6 | <LOQ | 1.46 | 0.73 | <LOD | 0.38 | <LOQ | 12.2 | <LOD | <LOQ | 1.44 | <LOQ | <LOD | 67.4 |
| Mean (ng/g dw) |  | <LOQ | 4.77 | <LOQ | 5.07 | 0.45 | 15.7 | 0.44 | 11.4 | 0.71 | 1.80 | 1.72 | <LOQ | 1.11 | 0.48 | 30.0 | <LOQ | 0.59 | 2.14 | <LOQ | <LOQ | 77.6 |
| s.d (ng/g dw) |  |  | 3.76 |  | 1.24 | 0.30 | 5.80 | 0.30 | 5.40 | 0.73 | 1.32 | 2.57 |  | 1.37 | 0.81 | 32.3 |  | 1.13 | 2.23 |  |  |  |
| min (ng/g dw) |  | <LOD | 2.09 | <LOQ | 3.76 | <LOQ | 6.71 | <LOQ | 4.94 | <LOQ | <LOQ | <LOD | <LOD | <LOQ | <LOD | 6.34 | <LOD | <LOD | <LOQ | <LOD | <LOD | 53.1 |
| max (ng/g dw) |  | <LOQ | 11.3 | 0.50 | 6.98 | 0.99 | 22.6 | 0.97 | 16.4 | 1.67 | 3.50 | 6.20 | 0.76 | 3.39 | 1.91 | 82.1 | <LOQ | 2.60 | 5.85 | <LOQ | <LOQ | 126 |
| Detect (%) | 0% | 20% | 100% | 100% | 100% | 100% | 100% | 100% | 100% | 100% | 100% | 80% | 20% | 100% | 60% | 100% | 40% | 60% | 100% | 60% | 40% |  |
|  |  |  |  |  |  |  |  |  |  |  |  |  |  |  |  |  |  |  |  |  |  |  |
| **Lagoon Sludge (n=18)** | |  |  |  |  |  |  |  |  |  |  |  |  |  |  |  |  |  |  |  |  |  |
| Median (ng/g dw) |  | <LOD | <LOQ | <LOD | <LOQ | <LOD | 0.25 | <LOQ | 0.33 | <LOQ | <LOQ | <LOD | <LOD | 0.13 | <LOD | 1.83 | 0.26 |  | <LOD |  |  | 5.03 |
| Mean (ng/g dw) |  | 0.57 | 0.37 | <LOQ | <LOQ | <LOQ | 0.54 | <LOQ | 0.50 | <LOQ | <LOQ | <LOQ | <LOQ | 0.23 | 0.28 | 1.97 | 1.60 |  | <LOQ |  |  | 7.12 |
| s.d (ng/g dw) |  | 1.28 | 0.46 |  |  |  | 0.85 |  | 0.56 |  |  |  |  | 0.25 | 0.63 | 0.98 | 2.77 |  |  |  |  |  |
| min (ng/g dw) |  | <LOD | <LOQ | <LOD | <LOD | <LOD | <LOD | <LOD | <LOQ | <LOD | <LOQ | <LOD | <LOD | <LOD | <LOD | 0.94 | <LOD |  | <LOD |  |  | 2.01 |
| max (ng/g dw) |  | 3.17 | 1.26 | 0.31 | 0.45 | <LOQ | 2.25 | <LOQ | 1.60 | <LOQ | 0.32 | <LOQ | <LOQ | 0.64 | 1.56 | 3.31 | 7.09 |  | <LOQ |  |  | 19.0 |
| Detect (%) | 0% | 33% | 100% | 33% | 83% | 50% | 83% | 67% | 100% | 83% | 100% | 33% | 50% | 83% | 33% | 100% | 83% | 0% | 17% | 0% | 0% |  |
|  |  |  |  |  |  |  |  |  |  |  |  |  |  |  |  |  |  |  |  |  |  |  |
| **Lagoon Dredge (n=3)** | |  |  |  |  |  |  |  |  |  |  |  |  |  |  |  |  |  |  |  |  |  |
| Mean (ng/g dw) | 1.53 | 0.60 | 2.68 | 2.53 | 15.5 | 0.70 | 0.90 | 0.43 | 0.45 | 0.42 | <LOQ | 0.90 | 0.40 | 10.3 | 0.73 | 57.6 | 2.97 | <LOQ | 0.30 | <LOQ | <LOD | 99.0 |
| s.d (ng/g dw) | 0.56 | 0.24 | 1.77 | 1.47 | 8.23 | 0.32 | 0.28 | 0.23 | 0.27 | 0.29 |  | 0.13 | 0.42 | 6.26 | 0.31 | 29.5 | 1.04 |  | 0.17 |  |  |  |
|  |  |  |  |  |  |  |  |  |  |  |  |  |  |  |  |  |  |  |  |  |  |  |

Distribution coefficients (log K_d_) were estimated for the compounds PFHxA, PFHxS, 6:2 FTS, PFOA, PFNA, PFOS, and PFDA where solid and aqueous concentrations were above the limit of quantitation using the same methods as Eriksson et al. (2017) and Sun et al. (2012). using the following formula:


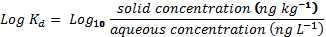


Calculated mean distribution coefficients in secondary sludge/effluent agreed with previously reported values in the studies for the compounds PFHxA, PFOA, PFNA and PFOS (Table S6). Between treatment plants, distribution coefficients varied for similar sample locations by up to 1.42 log units.

| Table S5. Distribution coefficient log K_d_ (L/Kg) for seven compounds calculated in primary sludge/effluent (PRI), secondary sludge/effluent (SEC) and lagoon sludge/effluent (LAGOON). Dash indicates not calculated. * indicates compounds where combined branched and linear concentration was reported. | | | | | | | | | |
| --- | --- | --- | --- | --- | --- | --- | --- | --- | --- |
| **WWTP-n** | | **Location** | **PFHxA** | **PFHxS** | **6:2 FTS** | **PFOA** | **PFNA** | **PFOS** | **PFDA** |
| WWTP-3 | | PRI | 1.42 | - | - | 1.85 | - | 2.73 | 3.10 |
| WWTP-3 | | SEC | 1.87 | - | 1.19 | 2.49 | - | 3.55 | 4.16 |
| WWTP-4 | | PRI | - | - | - | 1.48 | - | 2.60 | 3.40 |
| WWTP-4 | | SEC | 2.97 | 2.54 | 1.57 | 2.75 | - | 3.45 | 3.77 |
| WWTP-8 | | LAGOON | 0.47 | - | - | 1.31 | - | 3.35 | 2.96 |
| WWTP-9 | | PRI | 1.89 | - | 1.38 | 1.61 | 2.46 | 2.24 | 2.66 |
| WWTP-9 | | SEC | 2.00 | 2.27 | 2.07 | 2.37 | 2.86 | 3.46 | 3.67 |
| WWTP-10 | LAGOON | 1.52 | 1.74 | - | 1.02 | 1.62 | 2.15 | 2.38 |  |
| WWTP-16 | LAGOON | 1.96 | 2.31 | - | 1.78 | - | 2.41 | - |  |
| WWTP-17 | PRI | 2.14 | - | - | 1.50 | - | 2.45 | 1.81 |  |
| WWTP-17 | SEC | 2.03 | 1.46 | - | 2.06 | - | 2.77 | 3.15 |  |
| WWTP-18 | PRI | 1.69 | 1.74 | 0.74 | 1.74 | - | 2.66 | 2.26 |  |
| WWTP-18 | SEC | 2.03 | 1.78 | - | 2.24 | - | 2.83 | 2.94 |  |
|  |  |  |  |  |  |  |  |  |  |
| Mean | LAGOON | 1.32 | 2.02 | - | 1.37 | 1.62 | 2.64 | 2.67 |  |
| Mean | PRI | 1.78 | 1.74 | 1.06 | 1.64 | 2.46 | 2.54 | 2.65 |  |
| Mean | SEC | **2.22** | **2.09** | **1.61** | **2.42** | **2.86** | **3.31** | **3.69** |  |
|  |  |  |  |  |  |  |  |  |  |
| Erikkson 2017 | mean | 2.42 | 1.49 | 2.08 | 2.28 | 2.81 | 3.26 | 3.24 |  |
| Sun et al 2012 |  | ~2.4 | 3.09 | - | ~2.5 | 2.69 | 3.29 | ~3.1 |  |
|  |  |  |  |  |  |  |  |  |  |

| Table S6. Comparison of estimated daily PFAS discharged from WWTPs (g day^-1^) in Australia, Greece, Korea, Singapore and Spain. Data from Gallen et al. (2018) were calculated using WWTP flow information in supplementary material. | | | | | | | | |
| --- | --- | --- | --- | --- | --- | --- | --- | --- |
| **Country** | **∑(n)PFAS** | **Location** | **Year** | **Influent Types (Domestic, Industrial/ Trade Waste)** | **∑PFAS discharged** | **PFOS discharged** | **PFOA discharged** | **Reference** |
| Spain | 19 | Ebro River basin | 2010 | Dom | 66.9 | 9.02 | 0.062 | (Campo et al., 2014) |
|  | 19 |  | 2011 | Dom | 66 | 0.498 | 0.001 |  |
|  | 19 | Guadalquivir River basin | 2010 | Dom | 13.7 | 0.018 | 1.06 |  |
|  | 19 |  | 2011 | Dom | 32.2 | 0.009 | 0.286 |  |
|  | 19 | Jucar River basin | 2010 | Dom | 4 | 1.23 | 0.492 |  |
|  | 19 |  | 2011 | Dom | 16.9 | 0.403 | 2.39 |  |
|  | 19 | Llobregat River basin | 2010 | Dom/Ind | 10 | 1.09 | 1.80 |  |
|  | 19 |  | 2011 | Dom/Ind | 15.9 | 3.10 | 0.732 |  |
| Singapore | - | Plant B (CAS) | 2006/7 | 40% D, 60% I | - | 45 | 31 | (Yu et al., 2009) |
|  | - | Plant A (CAS Eff) | 2006/7 | 95% D, 5% I | - | 3.3 | 7.8 |  |
|  | - | Plant A (MBR Eff) | 2006/7 | 95% D, 5% I | - | 0.26 | 0.06 |  |
| Greece | 19 | Athens | 2010 | 80% D, 20% I | 65.2 | 9.39 | 11.6 | (Arvaniti et al., 2012) |
|  | 19 | Myteline | 2010 | 100% D | 2.02 | 0.016 | 0.019 |  |
| Korea | 15 | Domestic WWTPs (mean, n=5) | 2012 | >90% D | 51.1 | 3.8 | 14.4 | (Kim et al., 2012) |
|  | 15 | Industrial WWTPs (mean, n=5) | 2012 | >90% I | 27.3 | 1.4 | 3 |  |
|  | 15 | Mixed WWTPs (mean, n=5) | 2012 | 30-70% D, 30-70% I | 210.5 | 27.4 | 83.2 |  |
| Australia | - | QLD (ROC discharge) | 2008 | - | - | 0.9 | 1 | (Thompson et al., 2011) |
|  | 9 | Plant A | 2014 | 90% R; 8-10% T,I | 9.20 | 2.96 | 2.15 | (Gallen et al., 2018) |
|  | 9 | Plant B | 2014 | R, T/I | 0.79 | 0.13 | 0.25 |  |
|  | 9 | Plant C | 2014 | 85%R; 5%T; 10%G | 2.51 | 1.09 | 0.13 |  |
|  | 9 | Plant D | 2014 | 60% R; 30 % T,I; 10% G | 2.30 | 0.26 | 0.45 |  |
|  | 9 | Plant E | 2014 | R; T | 1.66 | 0.06 | 0.76 |  |
|  | 9 | Plant F | 2014 | 85% R; 15% T | 4.96 | 0.78 | 1.84 |  |
|  | 9 | Plant G | 2014 | R | 0.28 | 0.00 | 0.18 |  |
|  | 9 | Plant H | 2014 | 99.5% R; 0.5% T | 0.29 | 0.00 | 0.17 |  |
|  | 9 | Plant I | 2014 | 94.5% R; 5.5% T | 8.49 | 1.42 | 2.99 |  |
|  | 9 | Plant J | 2014 | 93.4% R; 0.6% T | 2.88 | 0.27 | 0.85 |  |
|  | 9 | Plant K | 2014 | 84% R; 15% I; 1% T | 0.33 | 0.06 | 0.09 |  |
|  | 9 | Plant L | 2014 | Primarily R. Also I, C, T | 0.05 | 0.01 | 0.01 |  |
|  | 9 | Plant M | 2014 | 66% R; 33% C; 1% I | 0.39 | 0.25 | 0.00 |  |
|  | 9 | Plant N | 2014 | R, T/I | 5.52 | 0.99 | 2.15 |  |
|  | 21 | WWTP-1 | 2017 | 5.7% TW | 0.29 | 0.06 | 0.05 | This study |
|  | 21 | WWTP-2 | 2017 | 8.9% TW | 6.7 | 1.6 | 0.35 |  |
|  | 21 | WWTP-3 | 2017 | 3% TW | 11 | 0.44 | 2.9 |  |
|  | 21 | WWTP-4 | 2017 | 16% TW | 12 | 2.6 | 1.3 |  |
|  | 21 | WWTP-5 | 2017 | 3.9% TW | 0.89 | 0.03 | 0.30 |  |
|  | 21 | WWTP-6 | 2017 | 3.7% TW | 0.39 | 0.01 | 0.15 |  |
|  | 21 | WWTP-7 | 2017 | 2% TW | 0.37 | 0.00 | 0.17 |  |
|  | 21 | WWTP-8 | 2017 | 4.9% TW | 0.11 | 0.00 | 0.02 |  |
|  | 21 | WWTP-9 | 2017 | 19% TW | 51 | 6.5 | 7.1 |  |
|  | 21 | WWTP-10 | 2017 | 9.5% TW | 0.16 | 0.04 | 0.02 |  |
|  | 21 | WWTP-11 | 2017 | 3.4% TW | 0.65 | 0.02 | 0.25 |  |
|  | 21 | WWTP-12 | 2017 | 4.1% TW | 1.1 | 0.00 | 0.08 |  |
|  | 21 | WWTP-13 | 2017 | 1.2% TW | 0.51 | 0.01 | 0.23 |  |
|  | 21 | WWTP-14 | 2017 | 4.7% TW | 0.05 | 0.00 | 0.01 |  |
|  | 21 | WWTP-15 | 2017 | 3% TW | 0.41 | 0.03 | 0.11 |  |
|  | 21 | WWTP-16 | 2017 | 1.8% TW | 0.17 | 0.01 | 0.03 |  |
|  | 21 | WWTP-17 | 2017 | 7.5% TW | 8.2 | 0.64 | 2.8 |  |
|  | 21 | WWTP-18 | 2017 | 6.5% TW | 19 | 1.1 | 3.4 |  |
|  | 21 | WWTP-19 | 2017 | 30% TW | 110 | 15 | 6.3 |  |

|  |  |
| --- | --- |
| Figure S1. Pearson correlation matrices for influent (left panel) and final effluent (right panel). Values display coefficient of correlation, crossed out circles were not significant (p>0.05). Pearson correlation coefficients were computed using the log_10_-transformed, pooled, influent and pooled final effluent data. | |

**References:**

ERIKSSON, U., HAGLUND, P. & KÄRRMAN, A. 2017. Contribution of precursor compounds to the release of per- and polyfluoroalkyl substances (PFASs) from waste water treatment plants (WWTPs). *Journal of Environmental Sciences,* 61**,** 80-90.

SUN, H., ZHANG, X., WANG, L., ZHANG, T., LI, F., HE, N. & ALDER, A. C. 2012. Perfluoroalkyl compounds in municipal WWTPs in Tianjin, China—concentrations, distribution and mass flow. *Environmental Science and Pollution Research,* 19**,** 1405-1415.
